# Obesity during pregnancy results in maternal intestinal inflammation, placental hypoxia, and alters fetal glucose metabolism at mid-gestation

**DOI:** 10.1038/s41598-019-54098-x

**Published:** 2019-11-26

**Authors:** Jessica G. Wallace, Christian J. Bellissimo, Erica Yeo, Yu Fei Xia, Jim J. Petrik, Michael G. Surette, Dawn M. E. Bowdish, Deborah M. Sloboda

**Affiliations:** 1 Department of Biochemistry and Biomedical Sciences, McMaster University, Hamilton, Canada; 2 Department of Medicine, McMaster University, Hamilton, Canada; 3 Department of Obstetrics and Gynecology, McMaster University, Hamilton, Canada; 4 Department of Pediatrics, McMaster University, Hamilton, Canada; 5 Department of Biomedical Sciences, University of Guelph, Guelph, Canada; 6 Department of Pathology and Molecular Medicine, McMaster University, Hamilton, Canada; 7 Farncombe Family Digestive Health Research Institute, McMaster University, Hamilton, Canada

**Keywords:** Intrauterine growth, Intrauterine growth, Reproductive biology, Reproductive biology

## Abstract

We investigated whether diet-induced changes in the maternal intestinal microbiota were associated with changes in bacterial metabolites and their receptors, intestinal inflammation, and placental inflammation at mid-gestation (E14.5) in female mice fed a control (17% kcal fat, n = 7) or a high-fat diet (HFD 60% kcal fat, n = 9; *ad libitum*) before and during pregnancy. Maternal diet-induced obesity (mDIO) resulted in a reduction in maternal fecal short-chain fatty acid producing *Lachnospiraceae*, lower cecal butyrate, intestinal antimicrobial peptide levels, and intestinal SCFA receptor *Ffar3, Ffar2* and *Hcar2* transcript levels. mDIO increased maternal intestinal pro-inflammatory NFκB activity, colonic CD3^+^ T cell number, and placental inflammation. Maternal obesity was associated with placental hypoxia, increased angiogenesis, and increased transcript levels of glucose and amino acid transporters. Maternal and fetal markers of gluconeogenic capacity were decreased in pregnancies complicated by obesity. We show that mDIO impairs bacterial metabolite signaling pathways in the mother at mid-gestation, which was associated with significant structural changes in placental blood vessels, likely as a result of placental hypoxia. It is likely that maternal intestinal changes contribute to adverse maternal and placental adaptations that, via alterations in fetal hepatic glucose handling, may impart increased risk of metabolic dysfunction in offspring.

## Introduction

The World Health Organization (WHO) reports that obesity rates across the globe have nearly tripled since 1975; greater than 1.9 billion adults are overweight and of these 650 million are clinically obese^1^. Of great concern, are the rates of obesity in women of reproductive age and children^2,3^. It is well recognized that maternal obesity and its associated obstetrical complications not only impact the health of the mother^4,5^, but also have significant impacts on the health of the offspring^6–8^. Maternal obesity is a key predictor of offspring obesity independent of genetic and lifestyle determinants^9^. Despite great advances in our understanding of the molecular and physiological pathways that govern the relationship between the early life environment and disease risk, the fundamental mechanisms underpinning the link between maternal and childhood obesity remain unclear.

Recent studies investigating host-microbial interactions have provided compelling evidence to suggest a role for the microbiota in mediating known hallmarks of obesity and metabolic disease including weight gain^10^, adiposity^10–13^, inflammation, and insulin insensitivity^11,14^. Aside from their established roles in the metabolism of polysaccharides, production of essential metabolites, and function of the gastrointestinal^15^ and immune^16^ systems, emerging evidence suggests the intestinal microbiota may influence maternal adaptations to pregnancy^17^. Although definitive evidence has yet to be uncovered^18^, preclinical models have shown that the colonization of germ free mice with third trimester human stool resulted in insulin resistance, suggesting that the maternal intestinal microbiota may be another factor that must now be considered when investigating early life adversity and fetal development.

As the maternal intestinal microbiota, and their metabolites, likely influence gut barrier function, as well as metabolic and immune adaptations during pregnancy, altered host-microbe interactions may impact placental development and function. How these interactions change in the context of obese pregnancies is unclear, although impaired placental function is known to contribute to an adverse early life environment that precipitates metabolic dysfunction in postnatal life. Thus, the aim of this study was to characterize the intestinal microbial changes that accompany maternal diet-induced obesity (mDIO) and to investigate whether mDIO was associated with altered short-chain fatty acid (SCFA) levels and their receptors, and maternal intestinal inflammation. Placental outcomes including markers of vascular development and inflammation, cellular markers of placental inflammation and downstream impacts on fetal hepatic gluconeogenesis, as a marker of metabolic capacity, were investigated.

## Results

### High-fat diet (HFD) intake before and during pregnancy results in maternal obesity but does not alter fetal or placental weight at E14.5

**Figure 1:**
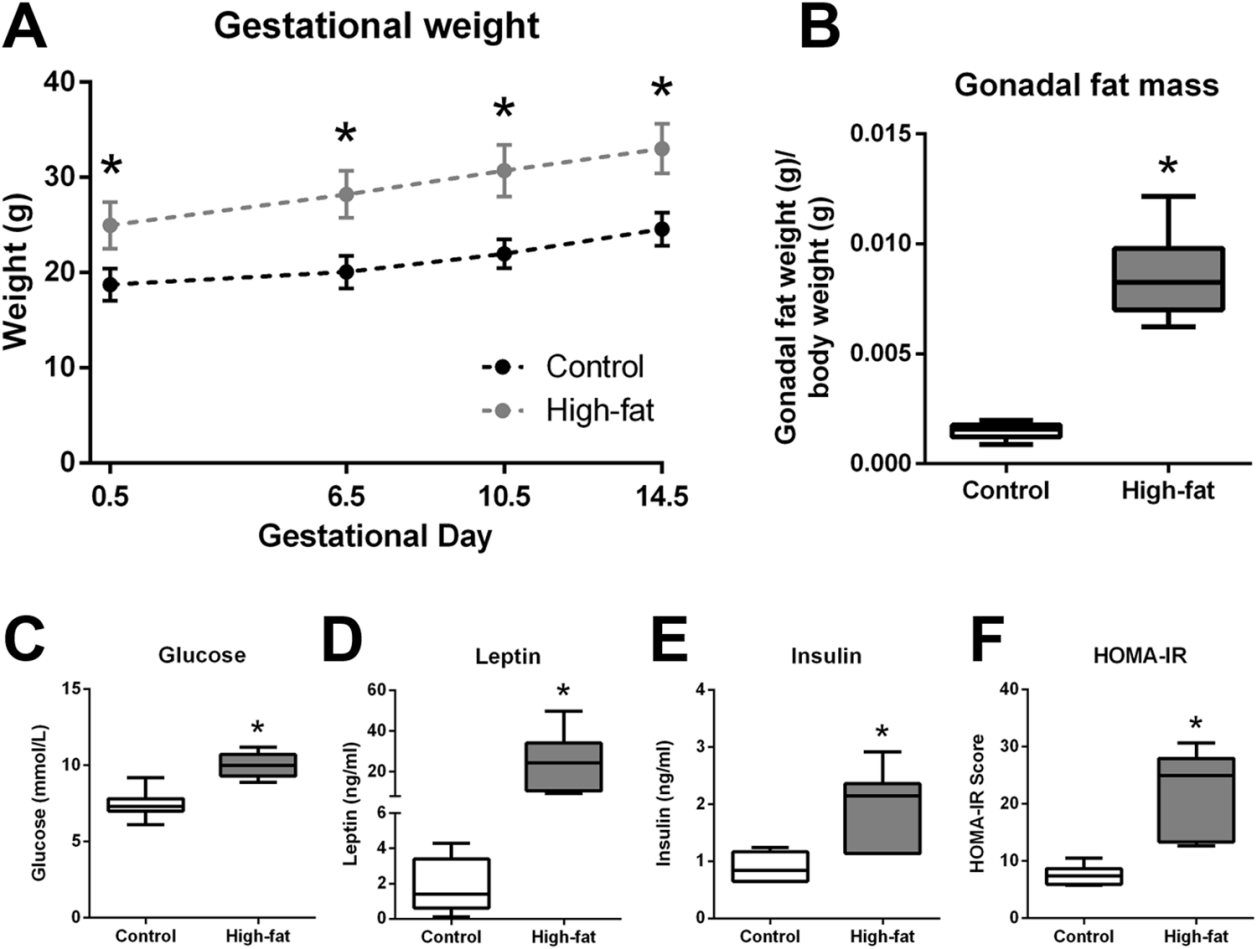
High-fat fed dams were heavier than controls at conception and throughout gestation and displayed increased adiposity, hyperglycemia, hyperleptinemia, hyperinsulinemia, and insulin resistance at E14.5. (**A**) Maternal body mass (g) during pregnancy in control (dark grey symbols, n = 7) and high-fat (light grey symbols, n = 9) fed dams. (**B**) Gonadal fat mass in control and high-fat fed dams. (**C**) Whole blood glucose in control and high-fat fed dams. (**D**) Serum leptin in control and high-fat fed dams. (**E**) Serum insulin in control and high-fat fed dams. (**F**) Homeostatic model assessment of insulin resistance (HOMA-IR) in control and high-fat fed dams. Data are presented as mean ± SEM (**A**) or box and whisker plots (**B**–**F**), min to max, where the centre line represents the median. Data were analyzed using a 2-way repeated measures ANOVA with Bonferroni’s *post-hoc* multiple comparisons and Student’s t-test where appropriate. *p < 0.05. Control: open boxes, (n = 7) and high-fat: grey boxes, (n = 9).

Consistent with our previous work^19,20^ and that of others^21–27^, high-fat fed females were significantly heavier than controls at mating and at all time points throughout gestation (Fig. 1A). As previously published^20^, caloric intake (kcal/g) was higher in high-fat fed mice early in pregnancy and normalized to control intake by E10.5 (data not shown). At E14.5, maternal gonadal fat mass was significantly higher in high-fat fed mothers compared to controls (Fig. 1B). Maternal whole blood glucose, serum leptin and insulin concentrations, and homeostatic model assessment of insulin resistance (HOMA-IR) were all higher in high-fat fed dams compared to controls (Fig. 1C–F). Fetal weights were similar between groups and remained unchanged even after controlling for fetal sex (Supplementary Fig. 2A). Placental weight was similar between groups (Supplementary Fig. 2B) and maternal high-fat diet did not impact litter size, sex ratio, or fetoplacental weight ratio (Supplementary Fig. 2A,B).

### Maternal microbial populations at E14.5 change with maternal diet-induced obesity

**Figure 2:**
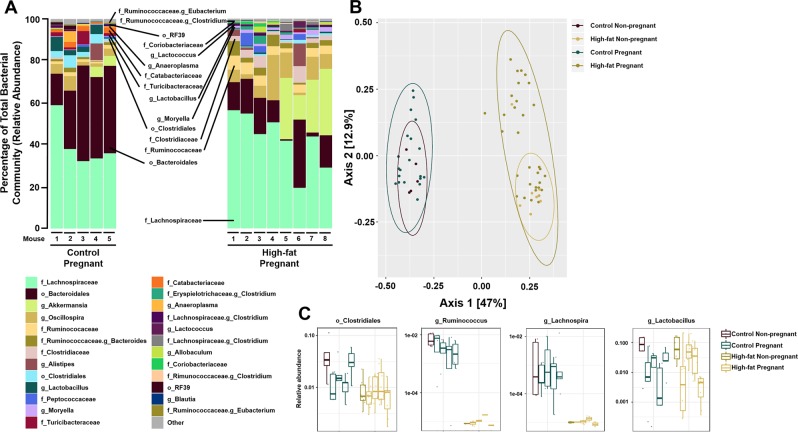
mDIO is associated with shifts in the maternal intestinal microbiota. (**A**) Taxonomic summaries of microbial relative abundance resolved to the order (o), family (f), or genus (g) level classification within pregnant control (n = 5) and high-fat (n = 8) females. (**B**) Principle Coordinate Analysis using the Bray-Curtis dissimilarity metric showed a significant separation of intestinal microbial communities as a result of diet but not pregnancy in control (n = 5) and high-fat (n = 8) females. (**C**) Relative abundance of *Clostridiales*, *Ruminococcus*, *Lachnospira*, and *Lactobacillus* in each female prior to pregnancy in control (maroon, n = 5) and high-fat (green, n = 8) and at four time points during gestation; E0.5, E6.5, E10.5, and E14.5 in control (teal, n = 5) and high-fat (beige, n = 8) females.

At E14.5, the overall relative abundance of 23 taxa were significantly different between control and high-fat dams (Fig. 2A). A significant separation in Bray-Curtis dissimilarity was identified as a result of diet (R^2^ = 0.453, p = 0.001) and pregnancy (R^2^ = 0.034, p = 0.009), where diet accounted for 45% of the among-sample variation and pregnancy accounted for 3.4% of the among-sample variation in both control-fed and high-fat fed dams (Fig. 2B). Most notable was the reduction in the relative abundance of known butyrate producing taxa *Clostridiales*, *Ruminococcaceae* and *Lachnospiraceae* with mDIO (Fig. 2C). Consistent with previous work^19,20^, we observed an impact of mDIO on pregnancy related shifts in the maternal intestinal microbiota (Supplementary Fig. 4A). Using the Bray Curtis dissimilarity metric, clustering by diet was identified in the ordination of Bray-Curtis dissimilarity and the effect of diet according to the PERMANOVA test was significant (p = 0.001) and substantially large (R^2^ = 0.598) explaining ~60% of the among-sample variation (Supplementary Fig. 4).

### mDIO reduces intestinal butyrate and β-defensin 3 and is associated with changes in maternal intestinal barrier proteins

**Figure 3:**
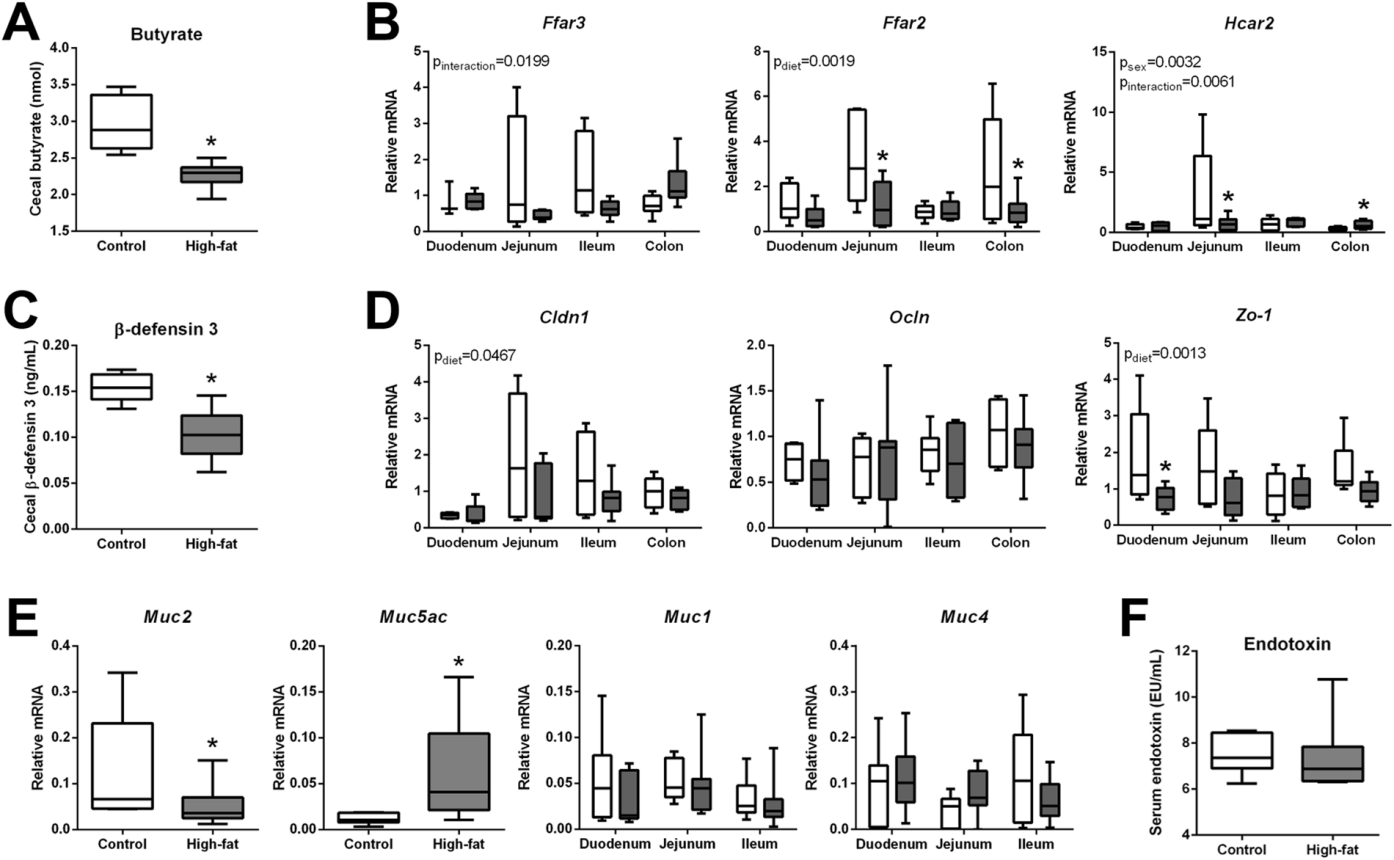
mDIO is associated with reduced maternal cecal butyrate and GPR levels, reduced β defensin-3 levels and altered mucin transcript levels. (**A**) Butyrate levels in control and high-fat dams. (**B**) Relative mRNA levels of *Ffar3*, *Ffar2*, and *Hcar2* in control and high-fat fed dams. (c) β defensin-3 peptide levels in control and high-fat fed dams. (**D**) Relative transcript levels of *Cldn1*, *Ocln*, and *Zo-1* in control and high-fat fed dams. (**E**) Relative transcript levels of intestinal mucin glycoproteins; *Muc2* and *Muc5ac* in the colon and *Muc1* and *Muc4* in the small intestine in control and high-fat fed dams. (**F**) Serum endotoxin in control and high-fat fed dams. Data are presented as box and whisker plots, min to max, where the centre line represents the median. Main effects are written as text in the figure. Data were analyzed with a 2-way ANOVA with Bonferroni’s *post-hoc* multiple comparisons or Student’s t-test where appropriate. *p < 0.05. Control: open boxes, (n = 7) and high-fat: grey boxes, (n = 9).

As mDIO was associated with a reduction in the relative abundance of intestinal taxa involved in the production of SCFA, we investigated whether this corresponded to reduced cecal SCFA levels. Consistent with a decrease in the relative abundance of butyrate producing taxa *Clostridiales*, *Lachnospiraceae*, *and Ruminococcaceae*, cecal butyrate levels were lower in high-fat fed dams compared to controls (Fig. 3A). Acetate, propionate, isobutyrate, isovalerate, pentanoate, and lactate cecal levels were not different between groups (data not shown). Maternal DIO altered intestinal transcript levels of SCFA receptors, *Ffar3* (p_interaction_ = 0.0199) and *Hcar2* (p_interaction_ = 0.0061) and reduced levels of *Ffar2* (p_diet_ = 0.0019) (Fig. 3B). Reductions in SCFAs receptor levels appeared to be predominantly located in the ileum and colon. A reduction in *Ffar2* is consistent with a decrease in intestinal antimicrobial peptide, β defensin-3 levels (Fig. 3C) as others have shown that *Ffar2*^*−/−*^ mice display lower levels of antimicrobial peptides^28^.

Butyrate has been shown to promote mucin 2 production in the colon^29–31^ and consistent with this, mRNA levels of the major secreted colonic *Muc2* were decreased, (Fig. 3E) although *Muc1* and *Muc4* were similar between groups (Fig. 3E). Colonic *Muc5ac* levels were significantly higher in mDIO mothers compared to controls (Fig. 3E). Butyrate has been shown to mediate intestinal barrier function through transcriptional regulation of tight junction protein claudin-1 and by the induction of occludin and ZO-1 redistribution in the cellular membrane^32^. Maternal DIO reduced claudin (*Cldn1* p_diet_ = 0.047) and *Zo-1* (p_diet_ = 0.0013) transcript levels (although only different in the duodenum), while occludin (*Ocln*) levels were unchanged (Fig. 3D). These observations are consistent with those that we have previously observed in the maternal intestine at term (E18.5) gestation^20^, but unlike our previously published data, we do not observe changes in maternal circulating levels of endotoxin with mDIO at E14.5 (Fig. 3F), suggesting that at midgestation, the level of maternal intestinal barrier compromise is not great enough to elicit increases in maternal serum endotoxin levels.

### mDIO is associated with increased intestinal NFκB activity and colonic CD3^+^ T cells

**Figure 4:**
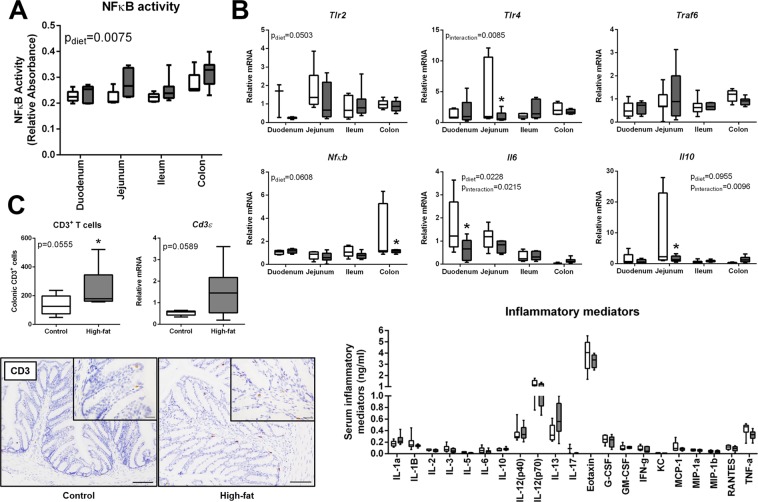
mDIO is associated with increased maternal intestinal NFκB activity and an increased number of colonic CD3^+^ T cells. (**A**) NFκB activity in the duodenum, jejunum, ileum, and colon tissue in control and high-fat dams. (**B**) Relative transcript levels of *Tlr2*, *Tlr4*, *Traf6*, *Nfκb*, *Il6*, and *Il10* in control and high-fat fed dams. (**C**) Number of colonic CD3^+^ T cells in control (open bars, n = 6) and high-fat (grey bars, n = 7) dams and relative transcript levels of colonic CD3ε in control and high-fat dams. Representative images of CD3 T cell immuno-localization in the colon of control and high-fat dams. Scale bar represents 100 µm. Maternal serum eotaxin, G-CSF, GM-CSF, IFN-γ, IL-1α, IL-1β, IL-2, IL-3, IL-4, IL-5, IL-6, IL-9, IL-10, IL-12 (p40), IL-12 (p70), IL-13, IL-17A, KC, MCP-1, MIP-1α, MIP-1β, RANTES, and TNF in control and high-fat fed dams. Main effects are written as text in the figure. Data were analyzed with a 2-way ANOVA with Bonferroni’s *post-hoc* multiple comparisons or Student’s t-test where appropriate. Control: open boxes, (n = 7) and high-fat: grey boxes, (n = 9). *p < 0.05.

Compromised intestinal barrier function is often accompanied by local inflammation and dysfunction of innate immune responses^33^. Maternal intestinal NFκB activity was significantly higher with mDIO (p_diet_ = 0.0075, Fig. 4A, control n = 6, mDIO n = 7) and thus we investigated key factors known to orchestrate intestinal pro-inflammatory signaling cascades in response to altered intestinal bacterial profiles^34,35^ during obesity. Consistent with our previous data in pregnant mice at E18.5, we found modest reductions in transcript levels of *Tlr2* (p_diet_ = 0.0500) and *Tlr4* (p_interaction_ = 0.0080) in the maternal intestine at E14.5 with mDIO (Fig. 4B). Downstream signaling component *Traf6* was unchanged and *Nfkb* (p_diet_ = 0.0608), and *Il6* (p_diet_ = 0.0230), transcript levels were modestly reduced with mDIO (Fig. 4B). *Il10* transcript levels were reduced in the jejunum with mDIO (p_interaction_ = 0.0096) (Fig. 4B). These observations are consistent with our observation that maternal serum levels of cytokines and chemokines (Fig. 4C) were similar between groups.

SCFAs are known to promote intestinal homeostasis through the immunomodulation of immune cell activation^10^, through G-protein-coupled receptor activation to regulate T cell differentiation to dampen inflammation^14^. We observed a modest increase in CD3^+^ T cell number in the maternal colon with mDIO (p = 0.0555) which was associated with a modest increase in *Cd3ε* transcript levels (p = 0.0589; Fig. 4C).

### mDIO promotes pro-inflammatory TLR signaling and induces a pro-inflammatory macrophage phenotype in the placenta

**Figure 5:**
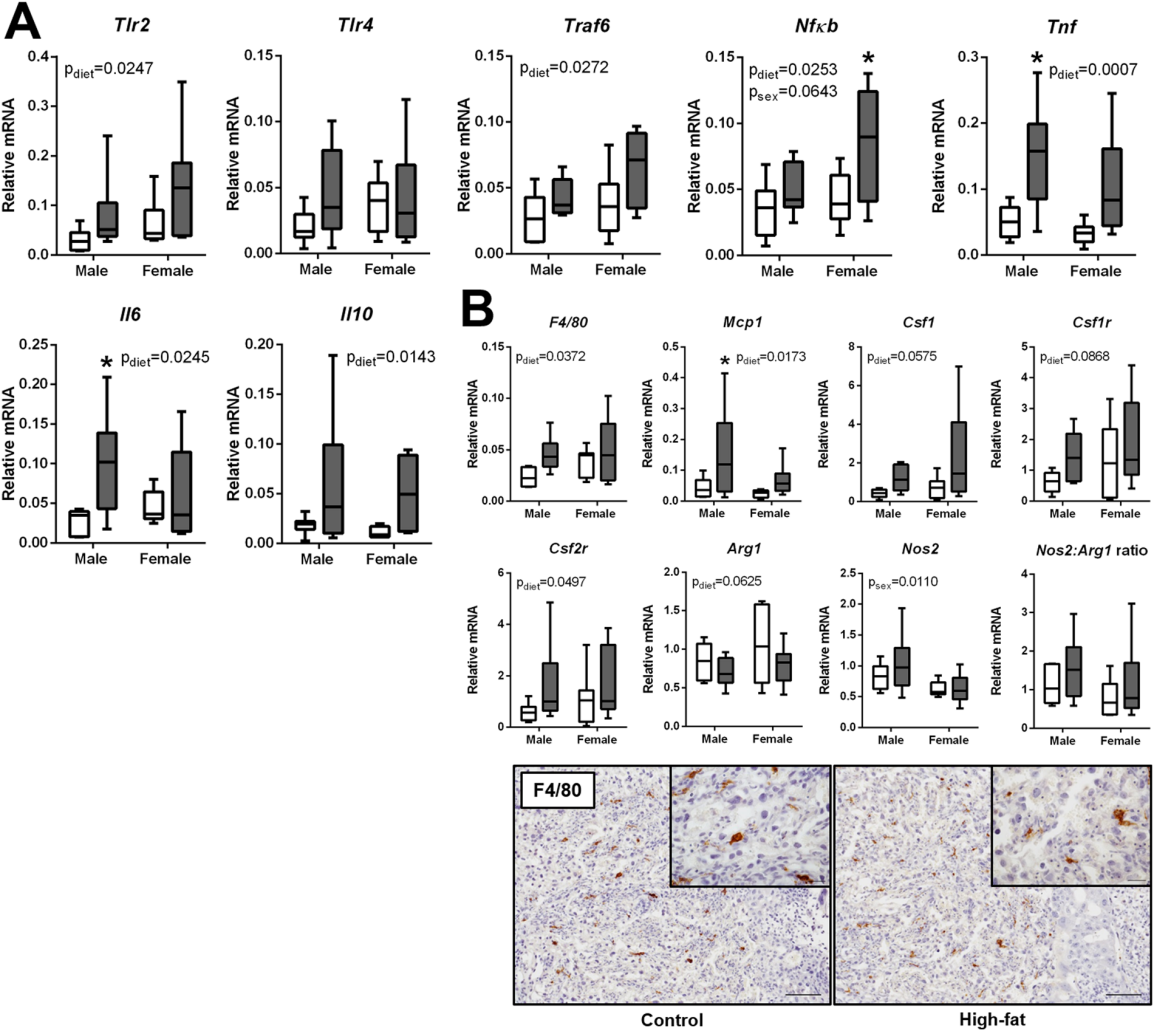
mDIO is associated with pro-inflammatory TLR signaling and a pro-inflammatory macrophage phenotype in the placenta. (**A**) Relative mRNA levels of toll-like receptor 2 (*Tlr2*), toll-like receptor 4 (*Tlr4*), TNF receptor-associated factor 6 (*Traf6*), nuclear factor kappa B (*Nfκb*), tumor necrosis factor (*Tnf*), interleukin 6 (*Il6*), and interleukin 10 (*Il10*) in placentae of control and high-fat dams. (**B**) Relative mRNA levels of pro-inflammatory macrophage cell markers, chemo-attractants, and polarization factors; *F4/80*, monocyte chemoattractant protein 1 (*Mcp1*), colony stimulating factor 1 (*Csf1*), colony stimulating factor receptor 1 (*Csf1r*), colony stimulating factor receptor 2 (*Csf2r*), arginase 1 (*Arg1*), nitric oxide synthase 2 (*Nos2*), and the ratio of *Nos2* to *Arg1* in placentae of control and high-fat dams. Representative images of F4/80 positive macrophage immuno-localization in the placenta of control and high-fat dams (females are shown). Scale bar represents 100 µm. Main effects are written as text in the figure. Data were analyzed with a 2-way ANOVA with Bonferroni’s *post-hoc* multiple comparisons test. Control: open boxes, (n = 7) and high-fat: grey boxes, (n = 9). *p < 0.05.

To determine whether mDIO was associated with altered inflammatory signaling in the placenta, we investigated transcript levels of key pattern recognition receptors, their downstream signaling components, and pro-inflammatory cytokines. mDIO resulted in an increase in both male and female placental *Tlr2* levels (p_diet_ = 0.0247), although *Tlr4* mRNA was unchanged (Fig. 5A). Both *Traf6* (p_diet_ = 0.0272) and *Nfkb *((p_diet_ = 0.0253, p_sex_ = 0.0643) mRNA levels were increased in mDIO placentae and this increase was more pronounced in placental tissue associated with female fetuses (Fig. 5A). *Tnf, Il6* and *Il10* mRNA levels were significantly increased with mDIO (Fig. 5A), while their receptor levels *Tnfr1a*, *Il6ra*, and *Il10ra* were similar between groups (data not shown).

To identify the cell types involved in pro-inflammatory signaling in the placenta with mDIO, we investigated transcript levels of cellular markers and chemo-attractants known to regulate pro-inflammatory macrophage function. Maternal DIO resulted in an increase in transcript levels of macrophage maturity marker F4/80 (*Adgre1)* (p_diet_ = 0.0372) and monocyte chemoattractant protein 1 (*Ccl2*) (p_diet_ = 0.0173), as well as a modest increase in macrophage colony stimulating factor 1 (*Csf1*) (p_diet_ = 0.0575) and *Csf2r* (p_diet_ = 0.0497). Arginase 1 (*Arg1*) mRNA levels, typically expressed by immunomodulatory macrophages involved in tissue remodeling and repair, were modestly reduced with mDIO (p_diet_ = 0.0625), although inducible nitric oxide synthase (*Nos2*) mRNA, expressed by pro-inflammatory macrophages, was unchanged, and the ratio of *Nos2* to *Arg1* was similar between groups (Fig. 5B). The number of F4/80 immunopositive macrophages was similar between groups (Fig. 5).

### mDIO promotes placental hypoxia and increases nutrient transporter transcript levels

**Figure 6:**
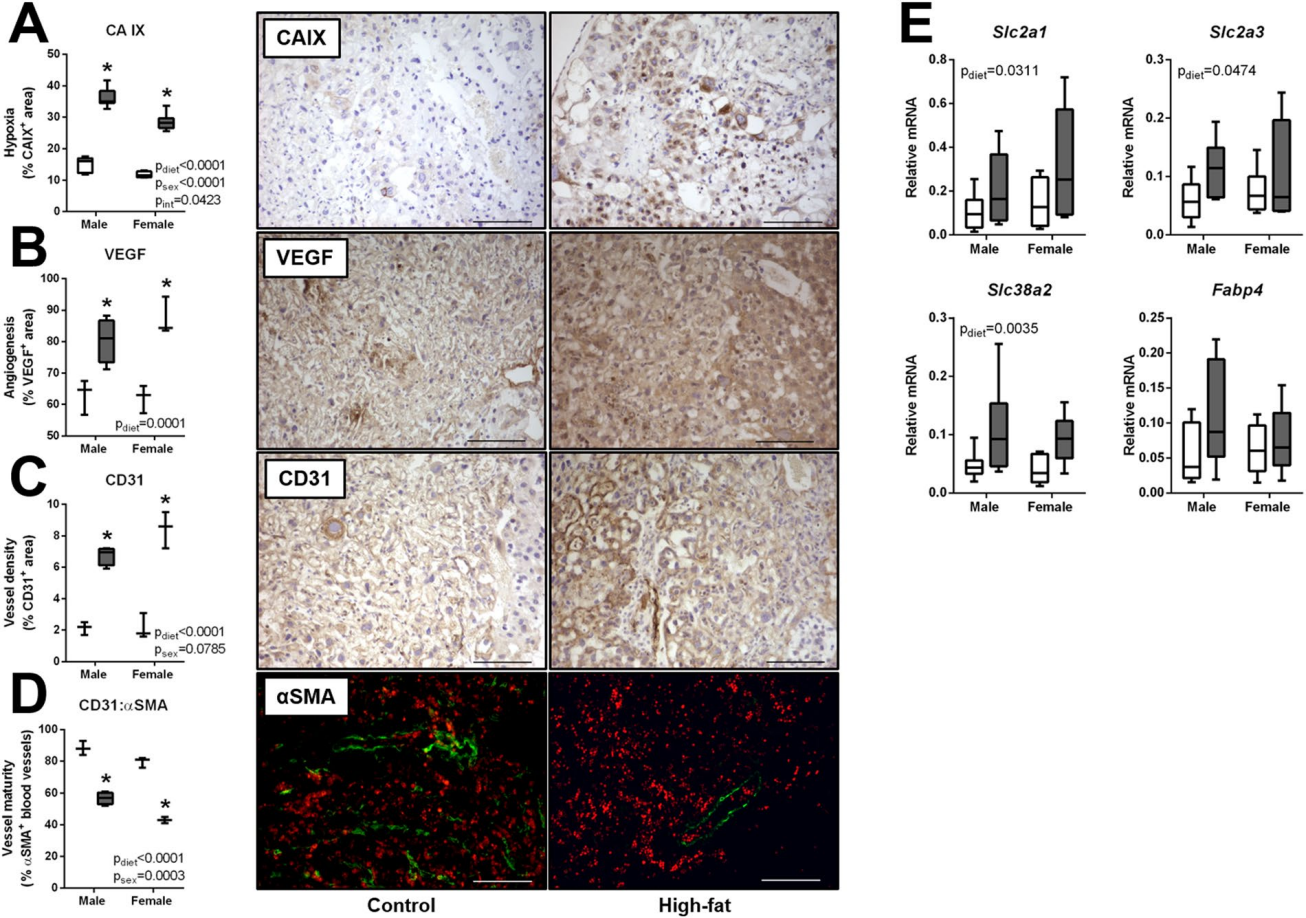
mDIO promotes placental hypoxia and angiogenesis, and alters nutrient transporters. Images represent immuno-localization of CAIX, VEGF and CD31 (brown staining) in control and high-fat dams. Scale bars represent 100 µm. (**A**) Carbonic anhydrase IX (CA IX) results from computerized image analysis of CAIX immunostaining in placentae of control (open bars, n = 3) and high-fat (grey bars, n = 4) dams. (**B**) Vascular endothelial growth factor results from computerized image analysis of VEGF immunostaining (open bars, n = 3) and high-fat (grey bars, n = 4) dams. (**C**) Cluster of differentiation (CD31) results from computerized image analysis of CD31 immunostaining in placentae of control (open bars, n = 3) and high-fat (grey bars, n = 4) dams. (**D**) Ratio of alpha-smooth muscle actin (α-SMA) to CD31 in placentae of control (open bars, n = 3) and high-fat (grey bars, n = 4) dams and representative images of α-SMA (green) pericytes lining CD31 (red) endothelial cells (**E**) Relative mRNA levels of glucose transporter 1 (*Slc2a1*), glucose transporter 3 (*Slc2a3*), system N/A amino acid transporter 2 (*Slc38a2*), and fatty acid binding protein 4 (*Fabp4*) in placentae of control and high-fat dams Control: open boxes, (n = 7) and high-fat: grey boxes, (n = 9). *p < 0.05.

Hypoxia^36^ and increased vascularization^20,37^ are previously reported placental outcomes as a result of mDIO. In the present study, carbonic anhydrase IX, a marker of hypoxia, was elevated in both male and female mDIO placentae (p_diet_ and p_sex = _0.001 and p_interaction_ = 0.04; Fig. 6A), where males showed modestly higher levels compared to females (Fig. 6A). Immunoreactive protein levels of pro-angiogenic vascular endothelial growth factor (VEGF) were elevated in both male and female mDIO placentae, (Fig. 6B). Consistent with a hypoxic, pro-angiogenic microenvironment, immunoreactive protein levels of platelet endothelial cell adhesion molecule 1 (CD31), an endothelial cell marker, were higher in both male and female mDIO placentae (Fig. 6C). We also investigated blood vessel maturity by immunolocalizing α-smooth muscle actin (α-SMA), a marker of placental pericytes. Interestingly, despite an apparent increase in placental CD31, both male and female placentae showed a reduction in immunoreactive α-SMA and a decrease in the ratio of CD31 to α-SMA suggesting reduced vessel maturity in mDIO placenta (Fig. 6D).

Since placental nutrient transport is critical for fetal growth, we also investigated transcript levels of nutrient transporters. Placental glucose transporter 1 (*Slc2a1*), glucose transporter 3 (*Slc2a3*), and system N/A amino acid transporter 2 (*Slc38a2*) mRNA levels were markedly elevated in mDIO placentae compared to controls. Levels of fatty acid binding protein 4 however (*Fabp4*) were similar between groups (Fig. 6E).

### Impacts of mDIO on maternal and fetal metabolic function

**Figure 7:**
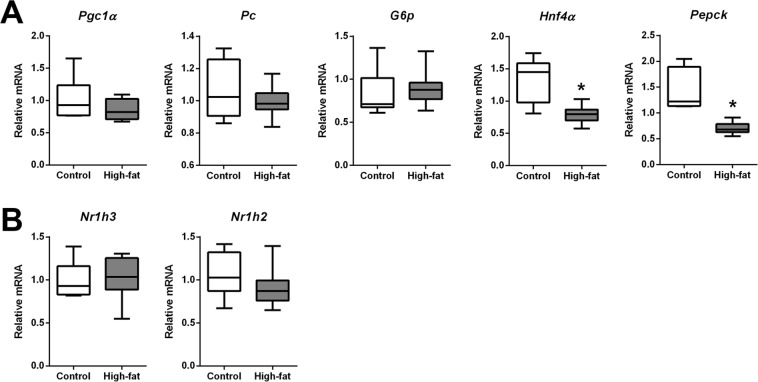
mDIO alters hepatic transcript levels of principle enzymes and transcription factors that regulate maternal hepatic gluconeogenesis. (**A**) Relative mRNA levels of; peroxisome proliferator-activated receptor gamma 1 alpha (*Pgc1α*), pyruvate carboxylase (*Pc*), glucose-6-phosphate (*G6p*), hepatocyte nuclear factor 4 alpha (*Hnf4α*), phosphoenolpyruvate carboxylase (*Pepck*) in the liver of control and high-fat dams. (**B**) Relative mRNA levels of liver X receptor alpha (*Nr1h3*) and liver X receptor beta (*Nr1h2*) in the liver of control and high-fat dams. Data are presented as box and whisker plots, min to max, where the centre line represents the median. Data were analyzed using a Student’s t-test. Control: open boxes, (n = 7) and high-fat: grey boxes, (n = 9). *p < 0.05.

Maternal adiposity is associated with impaired glucose homeostasis^38^, and obese women show both peripheral and hepatic insulin resistance, impaired insulin mediated hepatic glucose disposal^39^, and hyperlipidaemia^40^. We thus investigated the impacts of mDIO on principal enzymes known to regulate hepatic gluconeogenesis. Although maternal hepatic peroxisome proliferator activated receptor gamma 1 alpha (*Pgc1a*), pyruvate carboxylase (*Pc*), and glucose-6-phosphatase (*G6pc*) mRNA levels were not altered by mDIO, we found a significant reduction in transcript levels of cytosolic phosphoenolpyruvate carboxykinase (*Pepck*) and hepatic nuclear factor 4 alpha (*Hnf4a*), a known transcriptional activator of PEPCK in the maternal liver (Fig. 7A). The nuclear receptors liver X receptor (LXR)α and LXRβ are sensors of cholesterol metabolism and lipid biosynthesis and are known regulators of inflammatory cytokines, and suppress hepatic glucose production by inhibiting genes involved in gluconeogenesis including G6Pase (*G6pc*) and PEPCK (*Pepck*)^41,42^. LXRs are stimulated by fatty acid-induction of PPAR and by insulin^41^ and thus we investigated whether reduced *Pepck* mRNA levels were the result of LXR gene induction. Both LXRα (*Nr1h3)* and LXRβ *(Nr1h2)* transcript levels were unchanged with mDIO (Fig. 7B).

**Figure 8:**
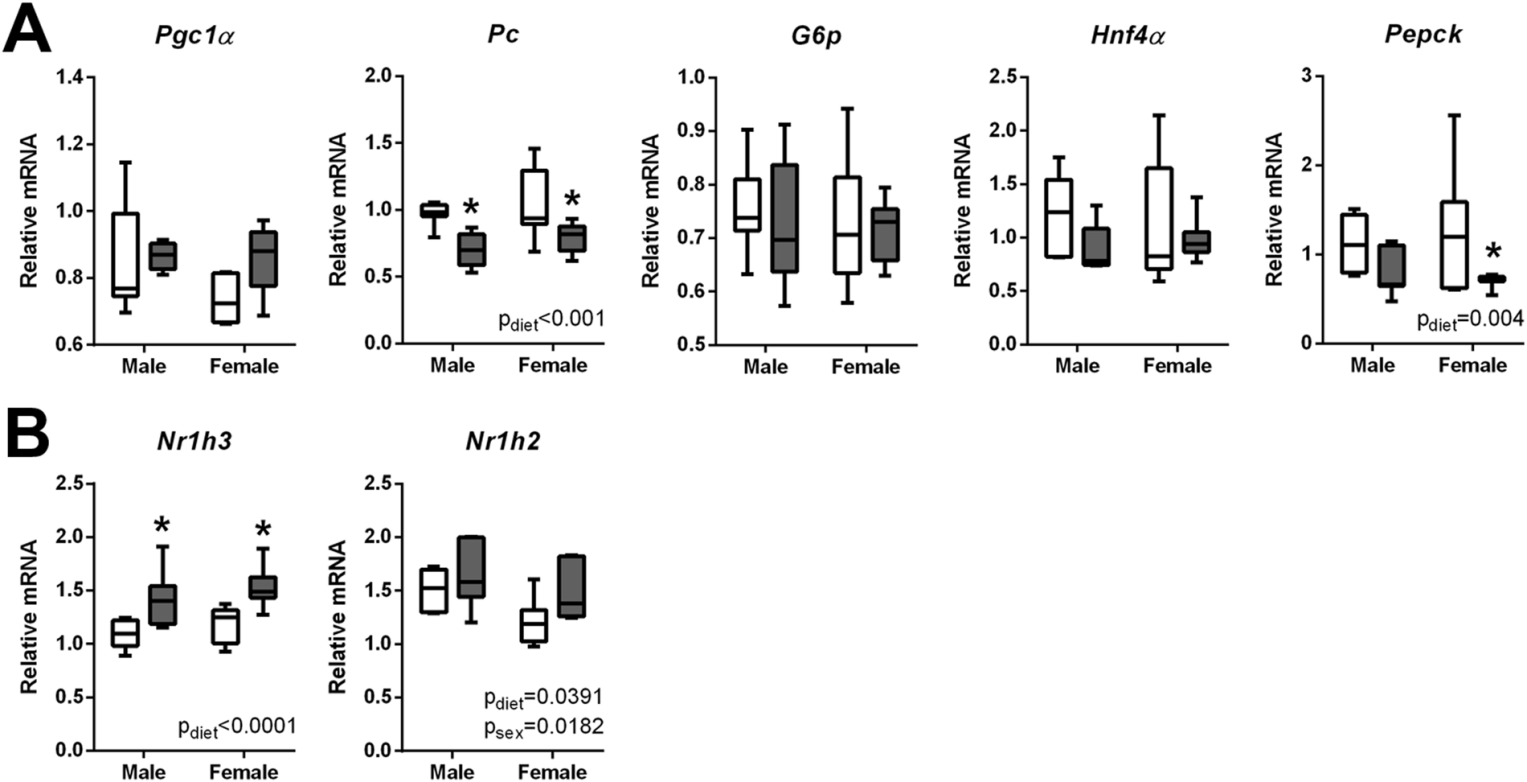
mDIO alters transcript levels of key enzymes and transcription factors that regulate fetal hepatic gluconeogenesis. (**A**) Relative mRNA levels of; peroxisome proliferator-activated receptor gamma 1 alpha (*Pgc1α*), pyruvate carboxylase (*Pc*), glucose-6-phosphate (*G6p*), hepatocyte nuclear factor 4 alpha (*Hnf4α*), phosphoenolpyruvate carboxylase (*pepck*) in the liver of control and high-fat exposed fetuses. (**B**) Relative mRNA levels of liver X receptor alpha (*Nr1h3*) and liver X receptor beta (*Nr1h2*) in the liver of control and high-fat exposed fetuses. Data are presented as box and whisker plots, min to max, where the centre line represents the median. Data were analyzed using a Student’s t-test. Control: open boxes, (n = 7) and high-fat: grey boxes, (n = 7) *p < 0.05.

Maternal obesity has known impacts on offspring metabolism^43^ which show a higher risk of insulin resistance^44,45^ and fatty liver disease^46,47^, suggesting that the developing liver is vulnerable to mDIO exposure. We found that both male and female hepatic pyruvate carboxylase (*Pc*) and PEPCK (*Pepck*) transcripts were significantly reduced in fetuses exposed to maternal obesity (Fig. 8A). This may be due to LXR-mediated suppression as both LXRα (*Nr1h3*) and LXRβ (*Nr1h2*) transcripts in male and female high-fat fetuses were increased (Fig. 8B). Transcript levels of *Pgc1α*, *G6pc*, and *Hnf4α* were similar between groups (Fig. 8C).

## Discussion

We have found that mDIO resulted in a reduction in the relative abundance of maternal SCFA producing gut microbial taxa, as well as corresponding decreases in maternal intestinal butyrate and SCFA receptor levels at mid-gestation in mice. mDIO resulted in increased maternal intestinal pro-inflammatory NFκB activity and an increased number of colonic CD3^+^ T cells, without associated changes in circulating soluble inflammatory factors. Furthermore, mDIO increased placental transcript levels of components of the TLR4 signaling pathway, inflammatory cytokines, macrophage cell markers, chemo-attractants, and factors known to regulate TNF-producing macrophage function. Placental inflammation was accompanied by hypoxia, increased vessel density and decreased maturity, both of which may impact nutrient transport. These maternal and placental changes were accompanied by altered fetal hepatic glucose handling, namely decreased gluconeogenic capacity.

We^19,20^ and others^17,48^ have shown that pregnancy is associated with a shift in the types and abundance of commensal bacteria in the maternal intestine. In the current study, maternal obesity significantly changed the abundance of Firmicutes, Bacteroidetes, Verrucomicrobia and Tenericutes phyla, similar to observations in male mouse models of obesity^12,13^, and in obese humans^49,50^. Metagenomic analyses in obese male mice, showed an increase in the representation of genes encoding proteins responsible for the synthesis of SCFAs from the fermentation of dietary carbohydrates^12^, utilized by the host for hepatic lipogenesis^11^. Whether this also applies to maternal intestinal microbiota during pregnancy is not clear. At E18.5, we showed that the gut microbiota of high fat fed pregnant mice was significantly enriched in genes involved in fatty acid and sulfur-containing amino acid metabolism, and glycolysis and gluconeogenesis compared to control fed pregnant mice^19^.

SCFAs produced by anaerobic bacteria play a key role in regulating intestinal barrier integrity^51,52^ but despite the widespread interest in SCFAs and GPRs and their role in obesity and inflammation, our understanding of their function during pregnancy remains unclear. In our study, obese dams had a decreased relative abundance of a number of bacterial genera belonging to the butyrate-producing families, and with this we observed a significant reduction in maternal caecal butyrate levels and decreased transcripts of SCFA receptors *Ffar3, Ffar2* and *Hcar2* and the mucosal protein *Muc2*. The extent to which these changes result in changes in maternal intestinal epithelial function is still unknown, although in other work we have observed an obesity-induced loss of maternal barrier integrity at E18.5^20^. Consistent with this observation, we show here that mDIO resulted in reductions in genes encoding tight junction proteins claudin-1 and ZO-1 in the maternal intestine, suggesting that during mDIO, SCFAs and their receptors may have lost their protective effects on maternal barrier function^51,52^. SCFA also regulate the production of intestinal antimicrobials via a GPR43-dependant pathway^28^. Here and in our previous work^19,20^ we show maternal obesity can disrupt this homeostasis via reductions in microbial communities that produce SCFAs, as well as their receptors potentially negatively affecting barrier proteins, mucosal proteins, and antimicrobial peptides (β-defensin 3).

SCFAs produced by anaerobic bacteria also play a key role in regulating intestinal immunity^53^. Colonic Treg cells regulate intestinal homeostasis and control inflammation by limiting proliferation of effector CD4^+^T cells. We show that maternal obesity resulted in a modest, but significant increase in the number of colonic CD3 + T cells and intestinal NFκB activity in all sections of the maternal intestine. This did not appear to be associated with increased transcript levels of either TLR2 or TLR4 signaling pathways and was also not associated with overall maternal serum inflammatory status. There are contrasting reports regarding maternal obesity and circulating markers of inflammation^54^ fueled by differences in species and in the timing of measures. We have previously reported that mDIO resulted in increased maternal serum LPS and TNF levels^20^ at E18.5 in mice, without changes in other pro-inflammatory cytokines. Similar results have been reported in term gestation obese women^55^. It is possible that maternal obesity-induced inflammatory status changes over the course of pregnancy, but this must be more thoroughly investigated.

Although we did not observe maternal systemic inflammation in mDIO dams we did observe increased markers of placental inflammation. This is consistent with studies in humans^56–58^, non-human-primates^59^, and ovine placentae^60^. Our data are limited to transcript levels, and do not describe immune cell specific changes, although we did investigate immune cell markers. We found that transcript levels of macrophage recruitment factor *Ccl2* and macrophage maturation marker *Adgre1* were elevated by mDIO, along with increases in macrophage activating cytokine expression (*Csf1*) and modest elevation in myeloid specific receptors *Csf1r* and *Csf2rα*. While our samples are highly enriched for placental tissue, we cannot exclude the possibility that changes in the abundance of macrophage-related transcripts were derived from changes to decidual macrophage populations from remaining decidual tissue, where tissue resident macrophages reside. Our ongoing studies will determine immunophenotypic changes to specific cells using more targeted methods including flow cytometry.

Alternatively activated macrophages are key regulators of normal placental angiogenesis^61^ and placental inflammation is strongly associated with impaired tissue function^60,62^. mDIO was associated with placental hypoxia and increased VEGF and CD31 immunostaining. Other studies show that obese human placenta have increased non-branching angiogenesis with increased VEGF immunostaining in non-villous cytotrophoblast and endothelial cells of intermediate and terminal villi, but decreased staining in syncytiotrophoblast^63^. Maternal obesity has been linked to placental vascular dysfunction^59,64,65^ but the independent effects of obesity on placental vascular development still remain to be fully clarified. Indeed, others have reported decreased vessel density in the context of mDIO^66,67^ using other surrogate endothelial markers. Placental hypervascularization has been described to occur in the context of hyperglycemia and hyperinsulinemia^68,69^, both of which are present in mDIO dams, but whether this is causative is unclear. Hypoxia is also a robust angiogenic stimulus capable of inducing changes to placental vasculature^42,70^. As such, further studies examining the relative contribution of these factors to placental development, and the etiology of placental hypoxia in the context of mDIO are warranted.

Placental adaptations to hypoxia likely fuel downstream changes in nutrient transport and our data are consistent with that of others^25,71^, showing that mDIO increased glucose (*Slc2a1*, *Slc2a3*) and amino acid (*Slc38a2*) transporter expression. This increase was accompanied by changes in markers of fetal glucose handling, where we observed a decrease in fetal gluconeogenic capacity that appears to be driven by increased fetal hepatic *Nr1h3* transcripts, and corresponding reductions in *Pepck* and pyruvate carboxylase transcript levels. LXRs are known regulators of adipogenesis, but also directly inhibit hepatic gluconeogenesis, including *Pepck* expression^42^. Fetal exposure to a maternal low protein diet in mice similarly resulted in reduced LXRα and LXRβ mRNA levels with corresponding changes in LXR gene promoter methylation^72^, an effect that appears to persist into early postnatal life in rats^73^. Postnatal modifications in LXR regulation are proposed to remove LXR’s brake on gluconeogenesis and result in postnatal glucose dysregulation^73^. Although the investigation of metabolism in offspring was beyond the scope of the current study, we have previously shown that a maternal high fat diet results in postnatal offspring insulin resistance and obesity^74^. The present study extends these observations and suggests that already at midgestation, fetuses are likely making critical hepatic adaptations in response to mDIO.

Accompanying these changes in fetal gluconeogenic capacity, we observed similar changes in maternal gluconeogenic capacity in dams that were fed a high fat diet, although these effects were far more modest. Seminal work in rats has shown that maternal gluconeogenic enzymes, their activity and the rate of *in vivo* gluconeogenesis increases with pregnancy^75^, thus maintaining a high maternal to fetal glucose ratio, facilitating feto-placental glucose transfer. Our observed reduction in maternal hepatic *Hnf4*α suggest perhaps that elevated maternal insulin levels, due to mDIO, may be acting through *Pgc1α / Hnf4α* pathways to reduce transcription of gluconeogenic target genes including *Pepck*. Although we did not observe changes in *Pgc1α* transcript levels, compromised activity of PGC-1α and HNF4 α have been associated with the development of insulin resistance and Type 2 diabetes^76–78^. It is unknown whether these changes in transcript levels of hepatic gluconeogenic regulators in the maternal liver persist postpartum, but it is interesting to note that maternal obesity during pregnancy is a risk factor for gestational diabetes^79,80^ and increased risk of postpartum Type 2 diabetes^80^. Future studies should set out to investigate not only the impacts of mDIO on the fetus and offspring but also on maternal metabolism in the postpartum period.

We recognize that our study is not without limitations. Due to the design of the study, we have only measured transcript levels of key markers, enzymes and signaling molecules. Although most of these are transcriptionally regulated, we are unable to comment on protein levels at the present time. Future studies should probe deeper into the impacts of mDIO on maternal hepatic glucose regulation and our fetal liver measures could be extended with measures of hepatic pyruvate, glycerol, and lipid content. We have previously published that mDIO results in reduced maternal intestinal barrier function^20^ but despite this, full investigation of maternal intestinal permeability is required to understand whether gut barrier function changes with advancing gestational age. Finally, a thorough investigation of maternal immunophenotyping using flow cytometry would improve our understanding of mDIO-induced changes in maternal immunity and its relationship to intestinal and placental inflammation.

## Conclusion

In conclusion, we show that maternal diet-induced obesity (mDIO) resulted in a reduction in the relative abundance of maternal short-chain fatty acid producing microbial genera, and corresponding decreases in maternal intestinal levels of the antimicrobial beta defensin, butyrate, and SCFA receptor levels at mid-gestation in mice. We found that mDIO increased maternal intestinal pro-inflammatory NFκB activity and increased number of colonic CD3^+^ T cells. These changes were accompanied by increased placental transcript levels of components of the TLR4 signaling pathway, inflammatory cytokines, macrophage cell markers, chemo-attractants, and factors known to regulate TNF-producing macrophages. Placental inflammation was associated with hypoxia, increased vessel density but decreased maturity, both of which may impact placental nutrient transport. These maternal and placental changes were accompanied by altered fetal hepatic gluconeogenic capacity. Maternal intestinal microbial metabolites and their receptors likely play a major role in mediating the impacts of maternal diet induced obesity. These maternal intestinal changes could contribute to adverse maternal and placental adaptations that, via alterations in fetal hepatic glucose handling, impart increased risk of metabolic dysfunction in the offspring.

## Materials and Methods

### Animal model

All animal procedures for this study were approved by the McMaster University Animal Research Ethics Board (Animal Utilization Protocol 12-10-38) in accordance with the guidelines of the Canadian Council of Animal Care. Female C57BL/6 J mice were obtained from the Jackson Laboratory (Bar Harbor, ME, Strain 000664), maintained in standard mouse cages in the same room with a constant temperature of 25 °C and a 12-h light, 12-h dark cycle, and fed a control standard diet (17% kcal fat, 29% kcal protein, 54% kcal CHO, 3 kcal/g; Harlan 8640 Teklad 22/5 Rodent Diet) and provided water *ad libitum*. Following a 1-week acclimation period, 8-week-old females were randomly assigned to 1 of 2 dietary interventions: control diet (Control n = 10; 17% kcal fat, 29% kcal protein, 54% kcal carbohydrate, 3.40 kcal/g; Harlan 8640 Teklad 22/5 Rodent Diet) or a high-fat diet (High-fat n = 10, 60% kcal fat, 20% kcal protein, 20% kcal carbohydrate, 5.24 kcal/g, Research Diets Inc., D12492). Females were housed two per cage with *ad libitum* food and water and were maintained on their respective diets for a total of 6 weeks. Body weight and caloric intake were recorded weekly.

After 6 weeks of dietary intervention, females were co-housed with control fed 8-week-old C57BL/6 J males (Jackson Laboratory, Bar Harbor, ME, Strain 000664) overnight and pregnancy confirmed by the observation of a vaginal plug and designated as embryonic (E) day 0.5. Pregnant females were maintained on their respective diets and water *ad libitum*. Throughout gestation maternal weight and caloric intake were recorded and maternal fecal pellets were collected at four gestational time points; E0.5, E6.5, E10.5 and E14.5 (Supplementary Fig. 1). Fecal pellets were stored at −80 °C until DNA extraction and sequencing was performed. Pregnant dams were sacrificed at late-mid gestational timepoint E14.5, at which time the murine placenta is fully developed. At E14.5, pregnant dams were fasted for 6 hours. Maternal body weight was recorded. Maternal whole blood glucose was measured using a commercial glucometer (Precision Xtra, Abbott Diabetes Inc.), a blood sample was collected, serum isolated, and stored at −80 °C until analyzed. Pregnant dams were sacrificed by cervical dislocation and gonadal fat pads were weighed as a proxy measure of maternal adiposity. Maternal cecal contents were collected, and sections of the maternal duodenum, jejunum, ileum, and colon were collected, flash frozen in liquid nitrogen and stored at −80 °C or fixed in 10% formalin. Placentae and fetuses were dissected, weights were recorded and whole placentae from half of each litter (randomly selected) were snap-frozen in liquid nitrogen or fixed in 4% paraformaldehyde. Fetal tails and livers were collected (to determine fetal sex and to investigate markers of fetal metabolic function respectively) and flash frozen in liquid nitrogen and stored at −80 °C until analyzed. All sample sizes in the study represent the dam as a biological replicate, with one female and one male fetus randomly selected from each litter for each tissue of interest.

### Maternal microbiota profiling: DNA extraction and 16S rRNA gene sequencing

Genomic DNA was extracted from fecal samples (control n = 5, high-fat n = 8) as previously described^19^, with minor modifications: 0.2 g of fecal material was mechanically lysed in 800 μl of 200 mM NaPO_4_ monobasic (pH 8) and 100 μl guanidinium thiocyanate-ethylenediaminetetraacetic-sarkosyl with 0.2 g of 0.1 mm glass beads (MoBio Laboratories Inc., Carlsbad, CA, USA). Additional enzymatic lysis was performed by adding 50 μl lysozyme (100 mg/ml) and 10 μl RNase A (10 mg/ml) and incubated for 1 hour at 37 °C, followed by the addition of 25 μl 25% sodium dodecyl sulfate, 25 μl proteinase K and 62.5 μl 5 M NaCl and was incubated for 1 hour at 65 °C. The resulting solution was centrifuged (12,000 g for 5 minutes). The supernatant was removed and combined with an equal volume of phenol:chloroform:isoamyl alcohol (25:24:1) in a new micro-centrifuge tube. The sample was vortexed and centrifuged (12,000 g for 10 minutes). The aqueous phase was removed and combined with 200 μl DNA binding buffer (Zymo Research, Irvine, CA, USA). This mixture was passed through a Clean and Concentrator-25 DNA column (Zymo Research, Irvine, CA, USA) according to kit directions, washed, and elution was performed with ultrapure water.

PCR amplification of the variable 3 (V3) region of the bacterial 16 S rRNA gene was performed on extracted DNA from each sample independently, as previously described^19^. Briefly, each reaction contained 5 pmol of primer, 200 mM of dNTPs, 1.5 μl 50 mM MgCl_2_, 2 μl of 10 mg/ml bovine serum albumin (irradiated with a transilluminator to eliminate contaminating DNA) and 0.25 μl Taq DNA polymerase (Life Technologies, Canada) for a total reaction volume of 50 μl. 341 F and 518 R rRNA gene primers were modified to include adapter sequences specific to the Illumina technology and 6-base pair barcodes were used to allow multiplexing of samples as described previously^81^. DNA products of the PCR amplification were subsequently sequenced using the Illumina MiSeq platform (2 × 150 bp) at the Farncombe Genomics Facility (McMaster University, Hamilton ON, Canada).

### Sequence processing and data analyses

As previously described^20,82^, the sl1p pipeline was used to process resultant FASTQ files^83^. In this pipeline, Cutadapt^84^ was used to trim primers and low quality bases, PANDASeq^85^ to align paired-end reads, AbundantOTU+^86^ to group reads into Operational Taxonomic Units (OTUs) based on 97% similarity, and the RDP Classifier^87^ as implemented in Quantitative Insights into Microbial Ecology (QIIME)^88^ against the Feb 4 2011 release of the Greengenes reference database^89^ to assign a taxonomy to each OTU. Any OTU not assigned to the bacterial domain was removed, as was any OTU to which only 1 sequence was assigned. Chimeric sequences were identified and removed with the usearch61 algorithm implemented in QIIME^90,91^. This processing resulted in a total of 7214096 reads (mean 11986 reads per sample; range: 60669–155790) and 2423 OTUs. All OTUs with a summed relative abundance of <1% across all samples were excluded. The data set used for analysis contained 3943882 reads (mean 110986 reads per sample; range 60669–155790) and 127 OTUs.

Analyses of these data were completed using R (v. 3.3.1,^92^) in R Studio^93^. Data were curated using the phyloseq (v. 1.19.1,^94^), dplyr (v. 0.7.4,^95^), and tidyr (v. 0.8.0,^96^) packages. Taxon relative abundance bar charts were generated using custom R scripts and ggplot2 (v. 2.2.1,). Measures of β-diversity were computed using phyloseq’s implementation of the Bray-Curtis dissimilarity metric on relative abundances. The proportion of among-community variation explained by maternal diet and by pregnancy was tested using the vegan (v. 2.4.4,) implementation of permutational multivariate analysis (PERMANOVA) in the adonis function. Groupings according to Bray-Curtis dissimilarity were visualized via Principal Coordinate Analysis (PCoA) ordination, implemented in phyloseq and plotted using phyloseq and ggplot2.

### Maternal cecal short chain fatty acids (SCFAs)

Maternal cecal SCFA levels were quantified as described previously^20^ with minor modifications, by the McMaster Regional Centre of Mass Spectrometry (MRCMS). Thirty mg of frozen cecal contents was acidified in a weight equivalent volume of 3.7% HCl. Ten μl of internal standards were added to each sample and SCFA extraction was performed by adding 500 μl of diethyl ether to each sample, and the resultant solution was vortexed for 15 minutes. After vortexing, 800 μl of diethyl ether cecal extract was transferred to a clean 1.5 ml centrifuge tube. A 60 μl aliquot of each diethyl ether cecal extract was derivatized with 20 μl of N-tert-butyldimethylsilyl-N-methyltrifluoroacetamide (MTBSTFA; Sigma-Aldrich) in a chromatographic vial containing an insert. The resultant organic extract-MTBSTFA mixture was incubated at ambient temperature for 1 hour and SCFA levels were quantified using a gas chromatograph (Agilent 6890 N GC), coupled to a mass spectrometer detector (Agilent 5973 N Mass Selective Detector).

### Maternal cecal β-defensin 3

Maternal β-defensin 3 levels were quantified in cecal contents using the Mouse β-defensin 3 ELISA kit (MyBioSource, San Diego, CA, USA) following the manufacturer’s instructions. Reading was performed using a plate reader at a wavelength of 450 nm (BioTek®, Oakville, Canada).

### Maternal intestinal NFκB activity

Maternal intestinal tissue was homogenized with ceramic beads using a Precellys 24 homogenizer at 5 m/s for 60 seconds in buffer containing 50 mM KH2PO4, 5 mM EDTA, 0.5 mM DTT, and 1.15% KCl with cOmplete protease inhibitor tablets (Roche Diagnostics, Canada). Protein concentrations were determined using the bicinchoninic acid (BCA) method in the intestinal supernatant after homogenates were centrifuged for 10 minutes at 10,000 g at 4 °C. NFκB activity was measured in maternal intestinal homogenate using the TransAM™ NFκB p65 Transcription Factor Assay Kit according to the manufacturer’s instructions (40096, Active Motif, Carlsbad, CA, USA).

### Immunohistochemistry and immunofluorescence

Formalin fixed maternal colon samples were processed, embedded in paraffin, and sectioned at 4 µm. Immuno-localization of CD3^+^ T cells in colonic sections was performed by the McMaster Immunology Research Centre John Mayberry Histology Facility with high pH antigen retrieval on the Leica Bond RX Rabbit protocol (1:150, Abcam, ab16669, RRID:AB_443425) and CD+ 3Tcells were counted in entire section, by an experimenter blinded to the study groups.

As previously described^20^, paraformaldehyde (4%) fixed placentae were processed, embedded in paraffin, and sectioned at 4 µm. Placental sections were immersed in 30% hydrogen peroxide to inhibit endogenous peroxidase activity for 10 minutes. Antigen retrieval was performed by incubating tissues in 10 mM sodium citrate buffer with Tween, pH 6.0, at 90 °C for 12 minutes. Nonspecific binding was blocked with 5% bovine serum albumin for 10 minutes at ambient temperature and incubated overnight at 4 °C with primary antibodies (Supplemental Table S1): carbonic anhydrase-IX (1:600, Abcam, ab15086, RRID:AB_2066533), VEGF (1:400, Santa Cruz Biotechnology, sc-152, RRID:AB_2212984), CD31 (1:200, Abcam, ab24590, RRID:AB_448167), or α-SMA (1:400, Santa Cruz Biotechnology, sc-32251, RRID:AB_262054). The following day, tissue sections were incubated at ambient temperature for 1–2 hours with anti-mouse, rat, or rabbit biotinylated or fluorescently conjugated secondary antibodies (Sigma-Aldrich, Canada and Vector Laboratories). For chromogenic IHC, tissues were incubated with ExtrAvidin (Sigma-Aldrich, Canada) or VectaStain horse-radish peroxidase-conjugated streptavidin (Vector Laboratories) for 1 hour. Antibody labelling was identified by chromogen detection with 3,3′-diaminobenzidine (DAB) (D4293; Sigma-Aldrich, Canada). All DAB stained sections were counterstained with Gills 2 hematoxylin (GHS216; Sigma-Aldrich, Canada). Measurements were taken in 6 fields of view per section at 250 × magnification. Image analysis was performed using an Olympus BX-61 microscope and integrated morphometry software (MetaMorph) (VEGF, CD31, αSMA, CAIX) or on a Nikon Eclipse NI microscope and Nikon NIS Elements Imaging Software (v.4.30.02) (CD3). The ratio of CD31 (endothelial cells) to αSMA (pericytes) positive area was used as a marker of vessel maturity. All image analyses were performed by an investigator blinded to the study groups.

### RNA extraction and cDNA synthesis

Total RNA was extracted from frozen maternal intestinal sections, liver, placentae, and fetal liver using TRIzol®-Reagent following the manufacturer’s instructions. Briefly, 45 mg of frozen tissue was homogenized in 900 μl TRIzol®-Reagent (Ambion, Life Technologies) and 0.2 g of glass beads (Sigma-Aldrich) and centrifuged at 12000 g for 10 minutes at 4 °C. The supernatant was removed and added to 300 μl chloroform. The solution was thoroughly mixed, incubated at ambient temperature for 3 minutes, and centrifuged at 12000 g for 10 minutes at 4 °C. The aqueous phase was removed, combined with 500 μl of isopropanol. The resultant solution was mixed thoroughly, incubated for 20 minutes at ambient temperature, and were centrifuged at 12000 g for 10 minutes at 4 °C. Total RNA was purified using RNeasy columns (Qiagen RNeasy ® Mini Kit) according to the manufacturer’s instructions. RNA was eluted from the RNeasy column following a 15-minute incubation with 20 μl of RNase free water heated to 55 °C. RNA quantity and quality were assessed using a NanoDrop 2000/2000c spectrophotometer (Thermo Scientific). For each sample, RNA concentration and integrity was >1.7. All RNA samples were stored at −80 °C until first strand complementary DNA (cDNA) synthesis. Following extraction, 2 μg of RNA was used for cDNA synthesis using the SuperScript® VILO™ cDNA synthesis kit (Life Technologies) according to the manufacturer’s instructions. Each reaction consisted of 4 μl of 5X VILO™ Reaction Mix, 2 μl of 10X SuperScript® Enzyme Mix, 4 μl of 250 ng/μl RNA and 12 μl of ultrapure water for a total reaction volume of 20 μl. The reactions were incubated in a thermocycler (Bio-Rad Laboratories, C1000 Touch) under the following cycling conditions: 25 °C for 10 minutes, 42 °C for 1 hour, and 85 °C for 5 minutes. Complementary DNA was stored at −20 °C until qPCR assays were performed.

### Quantitative polymerase chain reaction (qPCR)

To quantify transcript levels, quantitative PCR assays were performed using the LightCycler 480 II (Roche Diagnostics, Canada) and LightCycler 480 SYBR Green I Master Mix (Roche Diagnostics, Canada, 04887352001). Primers were designed using Primer-BLAST software available at NCBI (website: blast.ncbi.nlm.nih.gov) and manufactured by Life Technologies. Primer sequences are listed in Supplemental Table 2. The following PCR cycling conditions were used for each assay: enzyme activation at 95 °C for 5 minutes, amplification of the gene product through 40 successive cycles of 95 °C for 10 seconds, 60 °C for 10 seconds, and 72 °C for 10 seconds. Specificity of primers was tested by dissociation analysis and only primer sets producing a single peak were used. Each plate for a gene of interest contained a 10-fold serial dilution standard curve, generated using pooled cDNA. Each sample and standard curve point was run in triplicate. The crossing point (Cp) of each well was determined via the second derivative maximum method using LightCycler 480 Software, Release 1.5.1.62 (Roche Diagnostics, Canada). An arbitrary concentration was assigned to each well based on the standard curve Cp values by the software. The geometric mean of 2 or 3 housekeeping genes was used as reference gene value for each sample; β-Actin (*Actb)*, Cyclophilin (*Ppia)* and Hypoxanthine phosphoribosyltransferase (*Hprt*), β2 microglobulin (*B2M*), and Non-POU domain containing octamer (*Nono*). Relative mRNA levels for each sample were determined by dividing the geometric mean of the triplicate for each sample by that sample’s reference gene value.

### Serum biochemistry

#### Maternal serum insulin and leptin concentrations

Maternal serum insulin and leptin concentrations were measured using a Bio-Plex Pro™ Mouse Diabetes Standard 8-plex Assay kit (Bio-Rad Laboratories) on the Bio-Plex® 200 system (Bio-Rad Laboratories) using Bio-Plex Manager™ software (Bio-Plex Manager™ 6.1 © Copyright 2000 Bio-Rad Laboratories, Inc.). All standards, samples, and blanks were analyzed in duplicate and the mean value was determined accounting for dilution factors where appropriate. Only the samples which fell between the linear range of the standard curve were included in statistical analyses.

#### Maternal serum cytokine and endotoxin concentrations

Maternal serum cytokine concentrations were measured using the Bio-Plex Pro™ Mouse Cytokine 23-plex Assay kit (Bio-Rad Laboratories) following the manufacturer’s instructions. Reading was performed using a Bio-Plex® 200 system (Bio-Rad Laboratories) using Bio-Plex Manager™ software (Bio-Plex Manager™ 6.1 © Copyright 2000 Bio-Rad Laboratories, Inc.).

Maternal serum endotoxin concentrations were measured using the Pierce™ LAL Chromogenic Endotoxin quantitation kit (Thermo Fisher Scientific) following the manufacturer’s instructions. Reading was performed using a plate reader at a wavelength of 405 nm (BioTek®, Oakville, Canada).

### Statistical analyses

Maternal gestational weight was analyzed using a two-way repeated measures analysis of variance (2-way ANOVA), with maternal diet and gestational day as factors. Maternal intestinal measures were analyzed by 2-way ANOVA with diet and gut section as factors. Placental outcomes were analyzed by 2-way ANOVA with maternal diet and fetal sex as factors. A Bonferroni’s *post-hoc* multiple comparison test was used where the ANOVA was p < 0.05. All other data were analyzed using a two-tailed unpaired Student’s t-test where p < 0.05 was considered statistically significant. Within normally distributed data, outliers were removed using the Grubb’s test α = 0.05. Where appropriate, data are presented as box and whisker plots, where the whiskers represent the minimum and maximum, the box represents the 25^th^ and 75^th^ percentiles (lower and upper quartiles, respectively), and the centre line represents the 50^th^ percentile. All statistical analyses were performed using GraphPad Prism 6.0 (GraphPad Prism 6.0 © Copyright GraphPad Software Inc. 1994–2013) statistical software.

## Supplementary information

Supplemental information

## Data Availability

Data available upon request.

## References

[1] Obesity and overweight. (World Health Organization, 2015).

[2] Hedley AA (2004). Prevalence of overweight and obesity among US children, adolescents, and adults, 1999–2002. Jama.

[3] Ogden CL, Carroll MD, Kit BK, Flegal KM (2014). Prevalence of childhood and adult obesity in the United States, 2011-2012. Jama.

[4] Athukorala C, Rumbold AR, Willson KJ, Crowther CA (2010). The risk of adverse pregnancy outcomes in women who are overweight or obese. BMC pregnancy and childbirth.

[5] Crane JM, Murphy P, Burrage L, Hutchens D (2013). Maternal and perinatal outcomes of extreme obesity in pregnancy. Journal of obstetrics and gynaecology Canada: JOGC = Journal d’obstetrique et gynecologie du Canada: JOGC.

[6] Portela DS, Vieira TO, Matos SM, de Oliveira NF, Vieira GO (2015). Maternal obesity, environmental factors, cesarean delivery and breastfeeding as determinants of overweight and obesity in children: results from a cohort. BMC pregnancy and childbirth.

[7] Catalano PM, Ehrenberg HM (2006). The short- and long-term implications of maternal obesity on the mother and her offspring. BJOG: an international journal of obstetrics and gynaecology.

[8] Drake AJ, Reynolds RM (2010). Impact of maternal obesity on offspring obesity and cardiometabolic disease risk. Reproduction (Cambridge, England).

[9] Catalano P, deMouzon SH (2015). Maternal obesity and metabolic risk to the offspring: why lifestyle interventions may have not achieved the desired outcomes. International journal of obesity (2005).

[10] D’Souza WN (2017). Differing roles for short chain fatty acids and GPR43 agonism in the regulation of intestinal barrier function and immune responses. PloS one.

[11] Backhed F (2004). The gut microbiota as an environmental factor that regulates fat storage. Proceedings of the National Academy of Sciences of the United States of America.

[12] Turnbaugh PJ (2006). An obesity-associated gut microbiome with increased capacity for energy harvest. Nature.

[13] Turnbaugh PJ, Backhed F, Fulton L, Gordon JI (2008). Diet-induced obesity is linked to marked but reversible alterations in the mouse distal gut microbiome. Cell host & microbe.

[14] Kim CH, Park J, Kim M (2014). Gut microbiota-derived short-chain Fatty acids, T cells, and inflammation. Immune network.

[15] Thompson GR, Trexler PC (1971). Gastrointestinal structure and function in germ-free or gnotobiotic animals. Gut.

[16] Round JL, Mazmanian SK (2009). The gut microbiota shapes intestinal immune responses during health and disease. Nature reviews. Immunology.

[17] Koren O (2012). Host remodeling of the gut microbiome and metabolic changes during pregnancy. Cell.

[18] Singh S, Karagas MR, Mueller NT (2017). Charting the Maternal and Infant Microbiome: What Is the Role of Diabetes and Obesity in Pregnancy?. Current diabetes reports.

[19] Gohir W (2015). Pregnancy-related changes in the maternal gut microbiota are dependent upon the mother’s periconceptional diet. Gut microbes.

[20] Gohir W (2019). High-fat diet intake modulates maternal intestinal adaptations to pregnancy and results in placental hypoxia, as well as altered fetal gut barrier proteins and immune markers. The Journal of physiology.

[21] Paul HA, Bomhof MR, Vogel HJ, Reimer RA (2016). Diet-induced changes in maternal gut microbiota and metabolomic profiles influence programming of offspring obesity risk in rats. Scientific reports.

[22] Samuelsson AM (2008). Diet-induced obesity in female mice leads to offspring hyperphagia, adiposity, hypertension, and insulin resistance: a novel murine model of developmental programming. Hypertension (Dallas, Tex.: 1979).

[23] Caluwaerts S (2007). Diet-induced obesity in gravid rats engenders early hyperadiposity in the offspring. Metabolism: clinical and experimental.

[24] Nivoit P (2009). Established diet-induced obesity in female rats leads to offspring hyperphagia, adiposity and insulin resistance. Diabetologia.

[25] Jones HN (2009). High-fat diet before and during pregnancy causes marked up-regulation of placental nutrient transport and fetal overgrowth in C57/BL6 mice. FASEB journal: official publication of the Federation of American Societies for Experimental Biology.

[26] Kruse M (2013). High-fat intake during pregnancy and lactation exacerbates high-fat diet-induced complications in male offspring in mice. Endocrinology.

[27] Gupta A, Srinivasan M, Thamadilok S, Patel MS (2009). Hypothalamic alterations in fetuses of high fat diet-fed obese female rats. The Journal of endocrinology.

[28] Zhao Y (2018). GPR43 mediates microbiota metabolite SCFA regulation of antimicrobial peptide expression in intestinal epithelial cells via activation of mTOR and STAT3. Mucosal immunology.

[29] Hatayama H, Iwashita J, Kuwajima A, Abe T (2007). The short chain fatty acid, butyrate, stimulates MUC2 mucin production in the human colon cancer cell line, LS174T. Biochemical and biophysical research communications.

[30] Augenlicht L, Shi L, Mariadason J, Laboisse C, Velcich A (2003). Repression of MUC2 gene expression by butyrate, a physiological regulator of intestinal cell maturation. Oncogene.

[31] Burger-van Paassen N (2009). The regulation of intestinal mucin MUC2 expression by short-chain fatty acids: implications for epithelial protection. The Biochemical journal.

[32] Wang HB, Wang PY, Wang X, Wan YL, Liu YC (2012). Butyrate enhances intestinal epithelial barrier function via up-regulation of tight junction protein Claudin-1 transcription. Digestive diseases and sciences.

[33] Pastorelli L, De Salvo C, Mercado JR, Vecchi M, Pizarro TT (2013). Central role of the gut epithelial barrier in the pathogenesis of chronic intestinal inflammation: lessons learned from animal models and human genetics. Frontiers in immunology.

[34] Schirmer M (2016). Linking the Human Gut Microbiome to Inflammatory Cytokine Production Capacity. Cell.

[35] Jiang W (2015). Dysbiosis gut microbiota associated with inflammation and impaired mucosal immune function in intestine of humans with non-alcoholic fatty liver disease. Scientific reports.

[36] Fernandez-Twinn DS (2017). Exercise rescues obese mothers’ insulin sensitivity, placental hypoxia and male offspring insulin sensitivity. Scientific reports.

[37] Hayes EK (2012). Adverse fetal and neonatal outcomes associated with a life-long high fat diet: role of altered development of the placental vasculature. PloS one.

[38] De Souza LR (2016). Hepatic fat and abdominal adiposity in early pregnancy together predict impaired glucose homeostasis in mid-pregnancy. Nutrition & diabetes.

[39] Sivan E, Chen X, Homko CJ, Reece EA, Boden G (1997). Longitudinal study of carbohydrate metabolism in healthy obese pregnant women. Diabetes care.

[40] Catalano PM (2002). Downregulated IRS-1 and PPARgamma in obese women with gestational diabetes: relationship to FFA during pregnancy. American journal of physiology. Endocrinology and metabolism.

[41] Steffensen KR, Gustafsson JA (2004). Putative metabolic effects of the liver X receptor (LXR). Diabetes.

[42] Cao G (2003). Antidiabetic action of a liver x receptor agonist mediated by inhibition of hepatic gluconeogenesis. The Journal of biological chemistry.

[43] Zambrano E (2016). Maternal Obesity: Lifelong Metabolic Outcomes for Offspring from Poor Developmental Trajectories During the Perinatal Period. Archives of medical research.

[44] Mingrone G (2008). Influence of maternal obesity on insulin sensitivity and secretion in offspring. Diabetes care.

[45] Catalano PM, Presley L, Minium J, Hauguel-de Mouzon S (2009). Fetuses of obese mothers develop insulin resistance in utero. Diabetes care.

[46] Mouralidarane A (2015). Maternal obesity programs offspring non-alcoholic fatty liver disease through disruption of 24-h rhythms in mice. International journal of obesity (2005).

[47] Mouralidarane A (2013). Maternal obesity programs offspring nonalcoholic fatty liver disease by innate immune dysfunction in mice. Hepatology (Baltimore, Md.).

[48] Collado MC, Isolauri E, Laitinen K, Salminen S (2008). Distinct composition of gut microbiota during pregnancy in overweight and normal-weight women. The American journal of clinical nutrition.

[49] Ley RE, Turnbaugh PJ, Klein S, Gordon JI (2006). Microbial ecology: human gut microbes associated with obesity. Nature.

[50] Santacruz A (2010). Gut microbiota composition is associated with body weight, weight gain and biochemical parameters in pregnant women. The British journal of nutrition.

[51] Feng Y, Wang Y, Wang P, Huang Y, Wang F (2018). Short-Chain Fatty Acids Manifest Stimulative and Protective Effects on Intestinal Barrier Function Through the Inhibition of NLRP3 Inflammasome and Autophagy. Cellular physiology and biochemistry: international journal of experimental cellular physiology, biochemistry, and pharmacology.

[52] Diao, H., Jiao, A. R., Yu, B., Mao, X. B. & Chen, D. W. Gastric infusion of short-chain fatty acids can improve intestinal barrier function in weaned piglets. **14**, 4, 10.1186/s12263-019-0626-x (2019). 10.1186/s12263-019-0626-x PMC6359775 30761185

[53] Smith PM (2013). The microbial metabolites, short-chain fatty acids, regulate colonic Treg cell homeostasis. Science (New York, N.Y.).

[54] Madan JC (2009). Maternal obesity and markers of inflammation in pregnancy. Cytokine.

[55] Basu S (2011). Pregravid obesity associates with increased maternal endotoxemia and metabolic inflammation. Obesity (Silver Spring, Md.).

[56] Challier JC (2008). Obesity in pregnancy stimulates macrophage accumulation and inflammation in the placenta. Placenta.

[57] Sisino G (2013). Diabetes during pregnancy influences Hofbauer cells, a subtype of placental macrophages, to acquire a pro-inflammatory phenotype. Biochimica et biophysica acta.

[58] Roberts KA (2011). Placental structure and inflammation in pregnancies associated with obesity. Placenta.

[59] Frias AE (2011). Maternal high-fat diet disturbs uteroplacental hemodynamics and increases the frequency of stillbirth in a nonhuman primate model of excess nutrition. Endocrinology.

[60] Zhu MJ, Du M, Nathanielsz PW, Ford SP (2010). Maternal obesity up-regulates inflammatory signaling pathways and enhances cytokine expression in the mid-gestation sheep placenta. Placenta.

[61] Zhao, H., Kalish, F. S. & Wong, R. J. Hypoxia regulates placental angiogenesis via alternatively activated macrophages. **80**, e12989, 10.1111/aji.12989 (2018). 10.1111/aji.12989 29932269

[62] Aye IL (2014). Increasing maternal body mass index is associated with systemic inflammation in the mother and the activation of distinct placental inflammatory pathways. Biology of reproduction.

[63] Dubova EA (2011). Vascular endothelial growth factor and its receptors in the placenta of pregnant women with obesity. Bulletin of experimental biology and medicine.

[64] Redmer DA (2009). Fetoplacental growth and vascular development in overnourished adolescent sheep at day 50, 90 and 130 of gestation. Reproduction (Cambridge, England).

[65] Hayes EK (2014). Trophoblast invasion and blood vessel remodeling are altered in a rat model of lifelong maternal obesity. Reproductive sciences (Thousand Oaks, Calif.).

[66] Stuart TJ (2018). Diet-induced obesity alters the maternal metabolome and early placenta transcriptome and decreases placenta vascularity in the mouse. Biology of reproduction.

[67] Son JS (2019). Exercise prevents the adverse effects of maternal obesity on placental vascularization and fetal growth. The Journal of physiology.

[68] Lassance L (2013). Hyperinsulinemia Stimulates Angiogenesis of Human Fetoplacental Endothelial Cells: A Possible Role of Insulin in Placental Hypervascularization in Diabetes Mellitus. *The*. Journal of Clinical Endocrinology & Metabolism.

[69] Desoye G (2018). The Human Placenta in Diabetes and Obesity: Friend or Foe? The 2017 Norbert Freinkel Award Lecture. Diabetes care.

[70] Burton GJ, Reshetnikova OS, Milovanov AP, Teleshova OV (1996). Stereological evaluation of vascular adaptations in human placental villi to differing forms of hypoxic stress. Placenta.

[71] Rosario FJ, Kanai Y, Powell TL, Jansson T (2015). Increased placental nutrient transport in a novel mouse model of maternal obesity with fetal overgrowth. Obesity (Silver Spring, Md.).

[72] van Straten EM (2010). The liver X-receptor gene promoter is hypermethylated in a mouse model of prenatal protein restriction. American journal of physiology. Regulatory, integrative and comparative physiology.

[73] Vo TX, Revesz A, Sohi G, Ma N, Hardy DB (2013). Maternal protein restriction leads to enhanced hepatic gluconeogenic gene expression in adult male rat offspring due to impaired expression of the liver X receptor. The Journal of endocrinology.

[74] Howie GJ, Sloboda DM, Kamal T, Vickers MH (2009). Maternal nutritional history predicts obesity in adult offspring independent of postnatal diet. The Journal of physiology.

[75] Valcarce C, Cuezva JM, Medina JM (1985). Increased gluconeogenesis in the rat at term gestation. Life sciences.

[76] Hara K (2002). A genetic variation in the PGC-1 gene could confer insulin resistance and susceptibility to Type II diabetes. Diabetologia.

[77] Mootha VK (2003). PGC-1alpha-responsive genes involved in oxidative phosphorylation are coordinately downregulated in human diabetes. Nature genetics.

[78] Silander K (2004). Genetic variation near the hepatocyte nuclear factor-4 alpha gene predicts susceptibility to type 2 diabetes. Diabetes.

[79] Leddy MA, Power ML, Schulkin J (2008). The impact of maternal obesity on maternal and fetal health. Reviews in obstetrics and gynecology.

[80] Chu SY (2007). Maternal obesity and risk of gestational diabetes mellitus. Diabetes care.

[81] Bartram AK, Lynch MD, Stearns JC, Moreno-Hagelsieb G, Neufeld JD (2011). Generation of multimillion-sequence 16S rRNA gene libraries from complex microbial communities by assembling paired-end illumina reads. Applied and environmental microbiology.

[82] Wallace JG, Potts RH, Szamosi JC, Surette MG, Sloboda DM (2018). The murine female intestinal microbiota does not shift throughout the estrous cycle. PloS one.

[83] Whelan FJ, Surette MG (2017). A comprehensive evaluation of the sl1p pipeline for 16S rRNA gene sequencing analysis. Microbiome.

[84] Martin M (2011). Cutadapt removes adapter sequences from high-throughput sequencing reads. EMBnet. journal.

[85] Masella AP, Bartram AK, Truszkowski JM, Brown DG, Neufeld JD (2012). PANDAseq: paired-end assembler for illumina sequences. BMC bioinformatics.

[86] Ye Y (2011). Identification and Quantification of Abundant Species from Pyrosequences of 16S rRNA by Consensus Alignment. Proceedings. IEEE International Conference on Bioinformatics and Biomedicine.

[87] Wang Q, Garrity GM, Tiedje JM, Cole JR (2007). Naive Bayesian classifier for rapid assignment of rRNA sequences into the new bacterial taxonomy. Applied and environmental microbiology.

[88] Caporaso JG (2010). QIIME allows analysis of high-throughput community sequencing data. Nature methods.

[89] DeSantis TZ (2006). Greengenes, a chimera-checked 16S rRNA gene database and workbench compatible with ARB. Applied and environmental microbiology.

[90] Caporaso JG (2011). Global patterns of 16S rRNA diversity at a depth of millions of sequences per sample. Proceedings of the National Academy of Sciences of the United States of America.

[91] Edgar RC, Haas BJ, Clemente JC, Quince C, Knight R (2011). UCHIME improves sensitivity and speed of chimera detection. Bioinformatics (Oxford, England).

[92] Team, R. C. R: A language and environment for statistical computing (2013).

[93] Team, R. (2016).

[94] McMurdie PJ, Holmes S (2013). phyloseq: an R package for reproducible interactive analysis and graphics of microbiome census data. PloS one.

[95] Hadley Wickham, R. F., Henry, L. & Müller, K. (2017).

[96] Wickham, H., Henry, L. & Wickham, M. H. Package ‘tidyr’. (2019).

