# Supplementary material for: Obesity during pregnancy results in maternal intestinal inflammation, placental hypoxia, and alters fetal glucose metabolism at mid-gestation
Source: Sci Rep. 2019 Nov 26;9:17621. doi: 10.1038/s41598-019-54098-x (PMC6879619; doi:10.1038/s41598-019-54098-x)
Supplement: Supplementary file 1 — Supplemental information [file 41598_2019_54098_MOESM1_ESM.pdf]

Obesity during pregnancy results in maternal intestinal inflammation, placental hypoxia, and alters fetal glucose metabolism at mid-gestation

Jessica G. Wallace[1,7], Christian J. Bellissimo[1,7], Erica Yeo[1,7], Yu Fei Xia[1,7], Jim J. Petrik[5], Michael G. Surette[1,2,7], Dawn M.E. Bowdish[6], and Deborah M. Sloboda[1,3,4,7*]

[1]Department of Biochemistry and Biomedical Sciences, McMaster University, Hamilton, Canada
[2]Department of Medicine, McMaster University, Hamilton, Canada
[3]Department of Obstetrics and Gynecology, McMaster University, Hamilton, Canada
[4]Department of Pediatrics, McMaster University, Hamilton, Canada
[5]Department of Biomedical Sciences, University of Guelph, Guelph, Canada
[6]Department of Pathology and Molecular Medicine, McMaster University, Hamilton, Canada
[7]Farncombe Family Digestive Health Research Institute, McMaster University, Hamilton, Canada

Figures

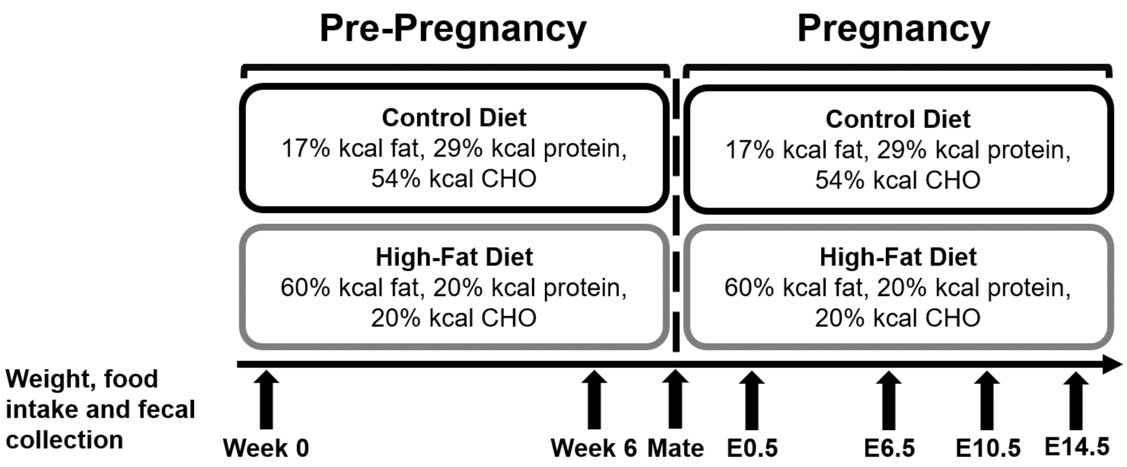

Supplemental figure S1. Experimental design. A schematic representation of the experimental animal design. C57BL/6 females were randomly assigned to 1 of 2 dietary groups: Control diet (17% kcal fat, 29% kcal protein, 54% kcal CHO, 3 kcal/g, n=10) or High fat diet (60% kcal fat, 20% kcal protein, 20% kcal CHO, 5.24 kcal/g, n=10) for 6 weeks and then mated with males. Fecal samples were collected from control females prior to dietary intervention (Week 0), after 6 weeks of dietary intervention (Week 6) and throughout gestation (E0.5, E6.5, E10.5 and E14.5, control n=7, high fat n=9). Abbreviations: CHO: Carbohydrate.

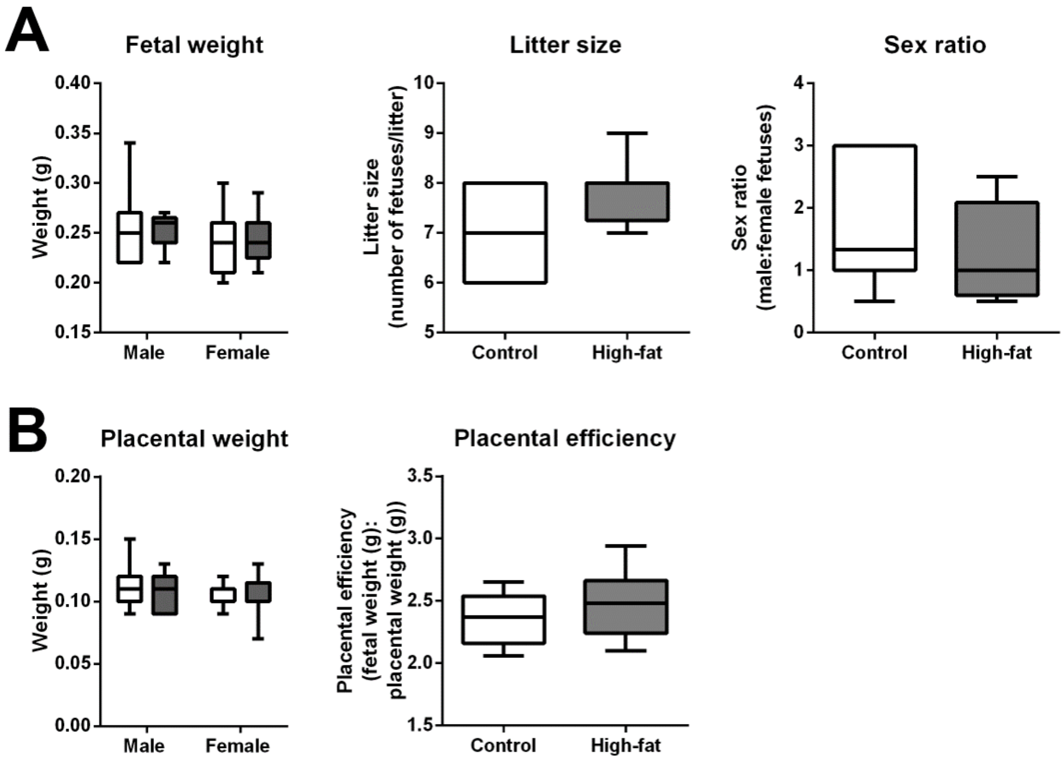

Supplemental figure S2. Maternal obesity did not impact fetal or placental weight at E14.5.
A. Fetal weight, litter size and sex ratio of control and high-fat exposed fetuses. **B.** Placental weight of control and high-fat exposed litters. Placental efficiency (feto-placental weight) of control and high-fat pregnancies. Data are presented as box and whisker plots, min to max, where the centre line represents the median. Data were analyzed using a Student's t-test. Control: open bar, n=7; high fat n= 9.

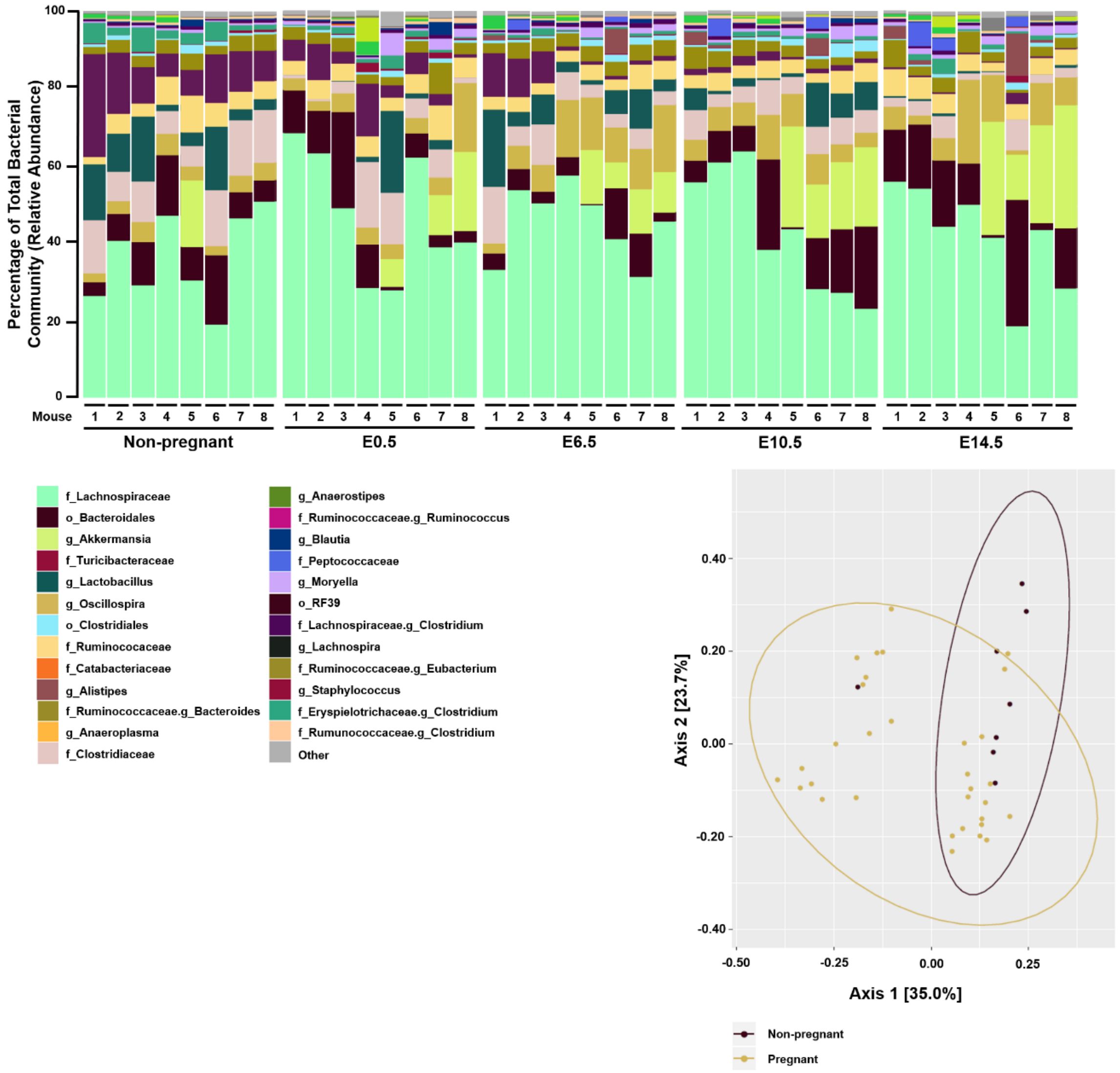

Supplemental figure S3. The maternal intestinal microbiota in control pregnancy.

A. Taxonomic summaries of microbial relative abundance resolved to the order (o), family (f), or genus (g) level classification within control fed females prior to pregnancy and at four time points during gestation; E0.5, E6.5, E10.5, and E14.5 (n=5). **B.** Principle Coordinate Analysis using the Bray-Curtis dissimilarity metric.

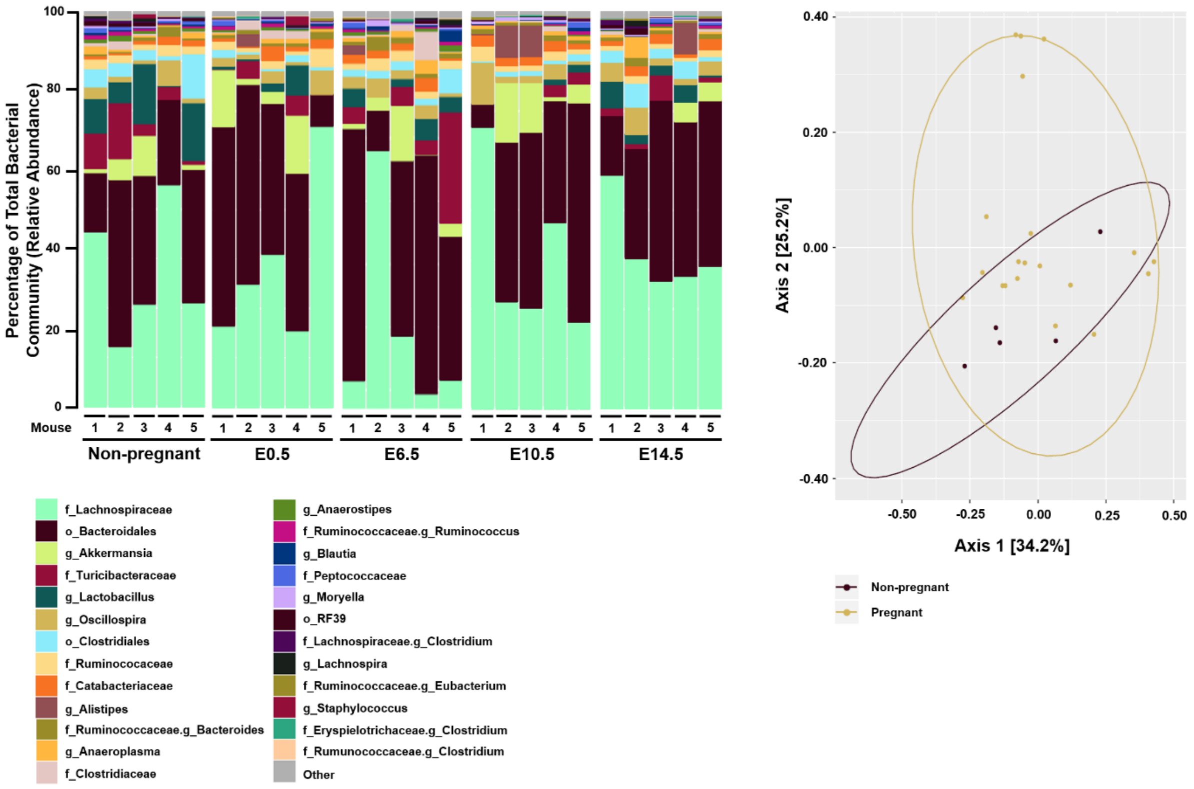

Supplemental figure S4. The maternal intestinal microbiota is remodeled by maternal obesity. A. Taxonomic summaries of microbial relative abundance resolved to the order (o), family (f), or genus (g) level classification within high-fat fed females prior to pregnancy and at four time points during gestation; E0.5, E6.5, E10.5, and E14.5 (n=8). We see a consistent mDIO impact on pregnancy related shifts in maternal intestinal microbiota **B.** Principle Coordinate Analysis using the Bray-Curtis dissimilarity metric. The effect of diet according to the PERMANOVA test was significant (p=0.001) and substantially large (R2=0.598).

Tables

Supplemental Table S1

**Primary antibodies**				
**Target peptide**	**Antibody name**	**Supplier**	**Catalogue number**	**Dilution**
**Intestine**				
CD3	Anti-CD3 antibody [SP7]	Abcam	ab16669	1:150
**Placenta**				
F4/80	Anti-F4/80 antibody [CI:A3-1]	Abcam	ab6640	1:100
CA IX	Anti-Carbonic Anhydrase IX antibody	Abcam	ab15086	1:600
VEGF	VEGF antibody [A-20]	Santa Cruz Biotechnology	sc-152	1:400
CD31	Anti-CD31 antibody [P2B1]	Abcam	ab24590	1:200
αSMA	A-Actin antibody [1A4]	Santa Cruz Biotechnology	sc-32251	1:400

Supplemental Table S2

Primer sequences

Gene	Forward primer	Reverse primer	Amplicon length (bp)	GenBank Accession Number
**Housekeeping**				
β-actin	AGATCAAGATCATTGCTCC	ACGCAGCTCAGTAACAGTC	174	NM_007393.5
Cyclophilin	CTTCGAGCTGTTTGCAGACA	TGGCGTGTAAAGTCACCAC	147	NM_008907.1
Hprt	CAGTCCCAGCGTCGTGATT	TCGAGCAAGTCTTTCAGTCC	142	NM_013556.2
β2m	CTCGGTGACCCTGGTCTTTC	TTGAGGGGTTTTCTGGATAGCA	71	NM_009735.3
Nono	GCCAGAATGAAGGCTTGACTAT	TATCAGGGGGAAGATTGCCCA	105	NM_001252518.1
**Inflammation**				
Ffar2	ACTTGCCCAAGGAGTTCTGG	AGCTGTCTGCTCTTCACCAC	59	NM_146187.4
Ffar3	GTCCAATACTCTGCATCTGTGAC	AGTCCCACGAGGAACACCAA	98	NM_001033316.2
Tlr2	AAGGAGGTGCGGACTGTTT	CCTCTGAGATTTGACGCTT	156	NM_011905.3
Tlr4	TCTGGGGAGGCACATCTTC	TGCTCAGGATTCGAGGCTT	72	NM_021297.3
Traf6	GCACGGAAACTTGGGTCTT	CTCTGTTGTCAGTCGACTTG	105	NM_009424.3
Nfκb	CTGCTCAGGTCCACTGCTTG	TTGCGGAAGGATGTCTCCA	112	NM_008689.2
Tnf	CAGACCCTCACACTCAGACTA	GGCTACAGGCTTGTCACTCG	86	NM_013693.3
Il6	GGGACTGATGCTGGTGACA	ACAGGTCTGTTGGGAGTGG	90	NM_001314054.1
Il10	ACTTTAAGGGTTACTTGGGTTGC	CCTGGGGCATCACTTCTACC	75	NM_010548.2
Cd3ε	CAGAAATGAAGTAATGAGCTGGCTG	CACTGTCTAGAGGGCACGTC	200	NM_007648.5
F4/80	TTGTGGTCCTAACTCAGTCTGC	AGACACTCATCAACATCTGCG	141	NM_010130.4
Mcp1	CCACAACCACCTCAAGCACT	AGGCATCACAGTCCGAGTCA	75	NM_011333.3
Csf1	TCTAGCCGAGATGTGGTGAC	TATGCGAAGGGGAAGCTCAC	195	NM_007778.4
Csf1r	AAGTGTCCTTAGAGGTGGTTTCC	GAAAGGGAACAGAGGAAGAGGC	104	NM_001037859.2
Csf2r	GAGTGACGTGCAGGAGGTTC	GAGGTCCTTCCTGAGGGTCT	116	NM_009970.2
Arg1	ACAAGACAGGGCTCCTTTCAG	GGCTTATGGTTACCCTCCCG	148	NM_007482.3
Nos2	GGTGAAGGGACTGAGCTGTT	ACGTTCTCCGTTCTCTTGCAG	103	NM_010927.4
**Barrier function**				
Muc1	CAAGTTCAGGTCAGGTCCG	TGACTTCACGTCAGAGGCAC	74	NM_013605.2
Muc4	AGATGGCTCTGAACCTAAGTATGC	AGGTGGTAGCCTTTGTAGCC	67	NM_080457.3
Muc2	GAAGCCAGATCCCGAAACCA	GAATCGGTAGACATCGCCGT	81	NM_023566.3
Muc5ac	CTGCCTGTACAATGGGACACT	AACATGTGTTGGTGCAGTCAGT	68	NM_010844.1
Cldn	TCTACGAGGGACTGTGGATG	TCAGATTCAGCAAGGAGTCG	84	NM_016674.4
Ocln	AGTTGAACTGTGGATTGGCAG	TAAGCGAACCTTGGCGGC	87	NM_008756.2
Zo-1	GAGAGACAAGATGTCCGCCA	CCATTGCTGTGCTCTTAGCG	50	NM_009386.2
**Placental nutrient transport**				
Slc2a1	GGCTTGCTTGTAGAGTGACG	TGTAGAACTCCTCAATAACCTTCTG	275	NM_011400.3
Slc2a3	AATAGGTAGGCTGGGCTTCG	AGAGATGGGGTCACCTTCGTT	184	NM_011401.4
Slc38a2	GCAGTGGAATCCTTGGGCTT	TAAAGATCCTCCTTCGTTGGCAG	140	NM_175121.3
Fabp4	AAGCTGGTGGTGGAATGTGTTA	CCTCTTCCTTTGGCTCATGC	77	NM_024406.2
**Gluconeogenesis**				
Pgc1α	TTGACTGGCGTCATTCGGG	AGAGCAGCACACTCTATGTCAC	84	NM_008904.2
Pc	AGGGCGGAGCTAACATCTACC	CCCCTCGAACTGTTTGGAACT	74	NM_001162946.1
G6p	TATTGTTGGTGCTGCCCTTG	AGTCCAATACATGAGGCTGGC	121	NM_175935.3
Pepck	CTGAAGGTGTCCCCCTTGTC	CATCTTGCCCTTGTGTTCTGC	110	NM_00110 44.2
Hnf4α	GGTAGGGGAGAATGCGACTC	TCAGATGGGGACGTGTCATTG	142	NM_008261.3
Nr1h3	CGACAGTTTTGGTAGAGGGAC	TGCAGAATCAGGAGAAACATCAG	106	NM_001177730.1
Nr1h2	CGCCACGTCACCCACTATTA	TCCTCTGGCTCCACGATGTA	96	NM_001285518.1
